# Targeting UXS1‐Dependent Glucuronate Detoxification Potentiates Metformin's Anti‐Tumor Efficacy in Lung Adenocarcinoma

**DOI:** 10.1002/advs.202510542

**Published:** 2026-05-10

**Authors:** Qihai Sui, Zhencong Chen, Guangyao Shan, Zhengyang Hu, Xing Jin, Jiaqi Liang, Yanjun Yi, Jiacheng Yin, Haochun Shi, Xifei Jiang, Junjie Xi, Zongwu Lin, Cheng Zhan, Fenghao Sun, Wei Jiang

**Affiliations:** ^1^ Department of Thoracic Surgery Zhongshan Hospital Fudan University Shanghai P. R. China; ^2^ Cancer Center Zhongshan Hospital Fudan University Shanghai P. R. China; ^3^ Zhangjiang Research Center Fudan University Shanghai P. R. China

**Keywords:** glucuronic acid metabolism, lung cancer, metformin, UGDH, UXS1

## Abstract

Despite the widely reported experimental anti‐tumor effects, metformin's role remains exceedingly complex, with contradictory results in clinical trials. Our study, based on metabolomics analysis of lung adenocarcinoma (LUAD) samples, xenografts, and cells, unveils a novel process that metformin promotes the conversion of UDP‐glucose (UDPG) to UDP‐glucuronic acid (UDPGA) in glucuronic acid metabolism. Mechanistically, metformin activates UDP‐glucose 6‐dehydrogenase (UGDH) through AMPK‐mediated phosphorylation of UGDH(S476), a previously unstudied phosphorylation site, impeding the binding of UDP‐Xyl to UGDH and the subsequent allosteric inhibition. Consequently, metformin‐treated cells are more reliant on UXS1, a downstream metabolic enzyme of UGDH, for detoxifying UDPGA based on the “kitchen‐sink” model. Through comprehensive virtual screening of a compound library, we identified that plantainoside is a potent UXS1‐targeting agent. Remarkably, when combined with metformin, plantainoside exhibits a superior synergistic lethal effect in LUAD cells, organoids, xenografts, and spontaneous models. Moreover, this combination not only directly targets tumor cells but also synergistically boosts CD8+ T cells and suppresses the differentiation of macrophages, thereby significantly enhancing immunotherapy efficacy. Collectively, our results shed light on metformin's complicated role by revealing its novel impact on glucuronic acid metabolism and dependence on UXS1; thus, targeting UXS1 combined with metformin represents a highly promising new strategy.

## Graphical abstract

This study reveals that metformin promotes glucuronic acid metabolism in lung adenocarcinoma by activating UGDH S476 phosphorylation and enhancing the conversion of UDPG to UDPGA based on metabolomics analysis. Through compound virtual screening, it is found that plantainoside targeting UGDH downstream UXS1 leads to UDPGA toxicity accumulation, resulting in synthetic lethality and immune activation.

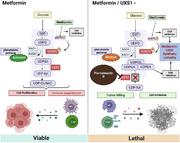

## 1 Introduction

Metformin, one of the most frequently prescribed medications for diabetes, has attracted considerable attention due to its anti‐inflammatory and anti‐tumor properties [1, 2, 3, 4]. Meanwhile, with the continuous acceleration of population aging, there is a growing number of cancer patients who are also diagnosed with diabetes, leading to an increasing application of metformin in this patient population [5, 6].

However, the therapeutic efficacy of metformin in cancer treatment remains unstable, with conflicting reports across various studies [7, 8, 9, 10]. For example, recent research findings suggest a potential adverse association between metformin use and the progression of type 2 diabetes in patients receiving immune checkpoint inhibitor therapy, indicating that metformin use may either have no effect or potentially increase the risk of disease progression and mortality in this patient population [11, 12, 13, 14]. Meanwhile, other studies reported that metformin might help lung cancer patients who are obese or overweight by improving their medical outcomes and making immunotherapy more effective for this growing group of patients [15, 16]. Hence, given the critical challenge of metformin's inconsistent antitumor efficacy in cancer patients, delving deeper into the complex mechanisms underlying metformin's varied efficacy in cancer patients and exploring strategies to enhance its antitumor effects is imperative.

In light of metformin's extensive regulation of cellular metabolism, we embarked on a study to pioneer novel strategies for amplifying its efficacy in cancer treatment from a metabolic perspective. Through metabolomic profiling of cells, mouse xenografts, and tumor samples from patients treated with metformin, we made the first‐ever discovery that metformin significantly promotes the generation of UDP‐glucuronic acid (UDPGA) from UDP‐glucose (UDPG). UDP‐glucose 6‐dehydrogenase (UGDH), the critical enzyme in glucuronate metabolism, catalyzes the conversion of UDPG to UDPGA and participates in vital metabolic processes [17, 18]. Subsequently, UXS1, the downstream metabolic enzyme of UGDH, converts UDPGA into UDP‐xylose (UDP‐Xyl), thereby preventing UDPGA accumulation and subsequent cellular damage [19, 20].

Our further investigation revealed that metformin phosphorylates UGDH via AMPK, the central sensor and regulator of metabolic stress, thereby stimulating UDPGA production and amplifying the dependency of metformin‐treated cells on UXS1. Capitalizing on this finding, we employed virtual screening combined with high‐content screening to identify and validate compounds that specifically target UXS1, which synergize with metformin to enhance its antitumor efficacy by not only directly targeting tumor cells but also counteracting immune evasion n through the “kitchen‐sink” model, thus offering a promising new strategy for cancer treatment.

## 2 Results

### 2.1 Metformin Facilitates the Metabolism of UDPG to UDPGA

**FIGURE 1:**
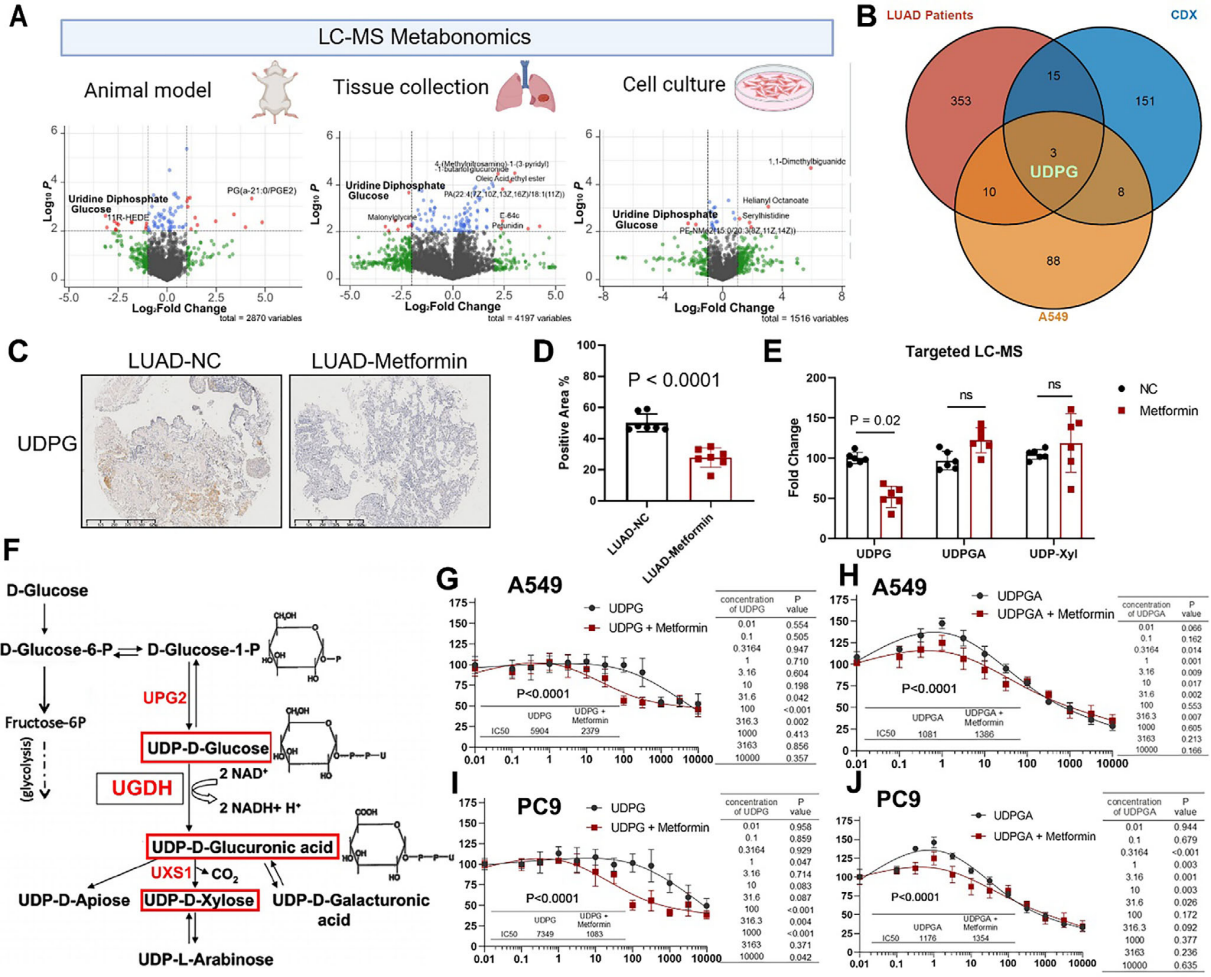
Metformin affects the glucuronide pathway. (A) Volcano plots showing the differentially abundant metabolites from metabolomic sequencing of tumors from LUAD patients with or without metformin intake (n = 6 per group), LUAD cell line A549 cultured with or without metformin (n = 6 per group), and subcutaneous tumors from nude mice treated with or without metformin (n = 6 per group). (B) Venn diagram illustrating that UDPG remains significantly altered across all three comparison groups. (C,D) Representative immunohistochemistry (IHC) images and quantitative analysis showing the differences in UDPG expression in LUAD patients with or without metformin intake (n = 7 per group). Scale bar: 625 µm. (E) Targeted mass spectrometry quantification verifying the differences in UDPG, UDPGA, and UDP‐Xyl levels in tumors of LUAD patients with or without metformin intake (n = 6 per group). (F) Schematic illustration of the main steps in the glucuronic acid pathway. (G‐J) CCK‐8 assays evaluating the effects of varying concentrations of UDPG and UDPGA (0.01, 0.1, 1, 10, 100, 1000, 10 000 mM) on the viability of A549 and PC9 cells, cultured with or without 1 mM metformin (n = 3 independent experiments). Statistical information: Statistical significance was determined using the two‐tailed unpaired Student's t‐test for comparisons between two groups (panels C, D, and E), and two‐way ANOVA followed by Bonferroni's post hoc test for multiple comparisons (panels G–J).

To clarify the effect of metformin on the metabolism of lung adenocarcinoma (LUAD), we used high‐throughput metabolomics to analyze the metabolite profiles in tumor tissues from LUAD patients, xenograft samples, and LUAD cells treated with metformin, and compared them with those from control tissues and cells that did not receive metformin treatment. Through intersection analysis, we observed a consistent and significant reduction in uridine diphosphate glucose (UDPG) levels across clinical samples, xenografts, and cells in the metformin‐treated groups (Figure 1A,B; Figure S1A and Table S1).

Multiple approaches were then utilized to validate the changes in UDPG levels. First, immunohistochemical (IHC) analysis was employed to visually demonstrate that UDPG levels were markedly lower in clinical tumor tissues from patients who had taken metformin compared to those who had not (Figure 1C,D). Additionally, we utilized targeted metabolomics to specifically measure the levels of metabolites related to the glucuronic acid pathway in tumor tissues from LUAD patients who did and did not receive metformin. The results indicated that UDPG levels were significantly reduced in patients taking metformin, while the levels of its downstream metabolites, UDPGA and UDP‐Xyl, showed slight increases that were not statistically significant (Figure 1E).

In the glucuronic acid pathway, glucose‐1‐phosphate reacts with uridine triphosphate (UTP) under the catalysis of uridine diphosphate glucose pyrophosphorylase 2 (UGP2) to generate UDPG. Subsequently, UDPG undergoes further catalysis by UGDH and UXS1, leading to the formation of UDPGA and UDP‐Xyl (Figure 1F).

Recent research has demonstrated that UDPG exhibits dose‐dependent inhibition in cells, while its downstream metabolite, UDPGA, further activates cell apoptosis. Our CCK‐8 experimental results reveal that low concentrations of UDPG (0–100 µM) have no discernible effect on LUAD cells (A549 and PC9), whereas low concentrations of UDPGA (0–10 µM) unexpectedly promote their proliferation (Figure 1G–J). However, at higher concentrations, both UDPG and UDPGA, particularly the latter, significantly impair the growth of LUAD (Figure 1G–J). However, no significant effect was shown when UDP‐Xyl was added (Figure S1B,C).

Meanwhile, although metformin did not alter the effect of low‐concentration UDPG, it significantly enhanced the toxic effect of high‐concentration UDPG on cells (Figure 1G–J). Additionally, metformin markedly inhibited the proliferative effect of low‐concentration UDPGA but did not significantly enhance the toxic effect of high‐concentration UDPGA (*p*< 0.001, Figure 1G–J). These results suggest that metformin may facilitate the metabolism of UDPG to UDPGA. Consequently, an increase in toxicity was observed.

### 2.2 Metformin Enhances the Glucuronic Acid Metabolism Through AMPK‐Mediated Phosphorylation of UGDH (S476)

**FIGURE 2:**
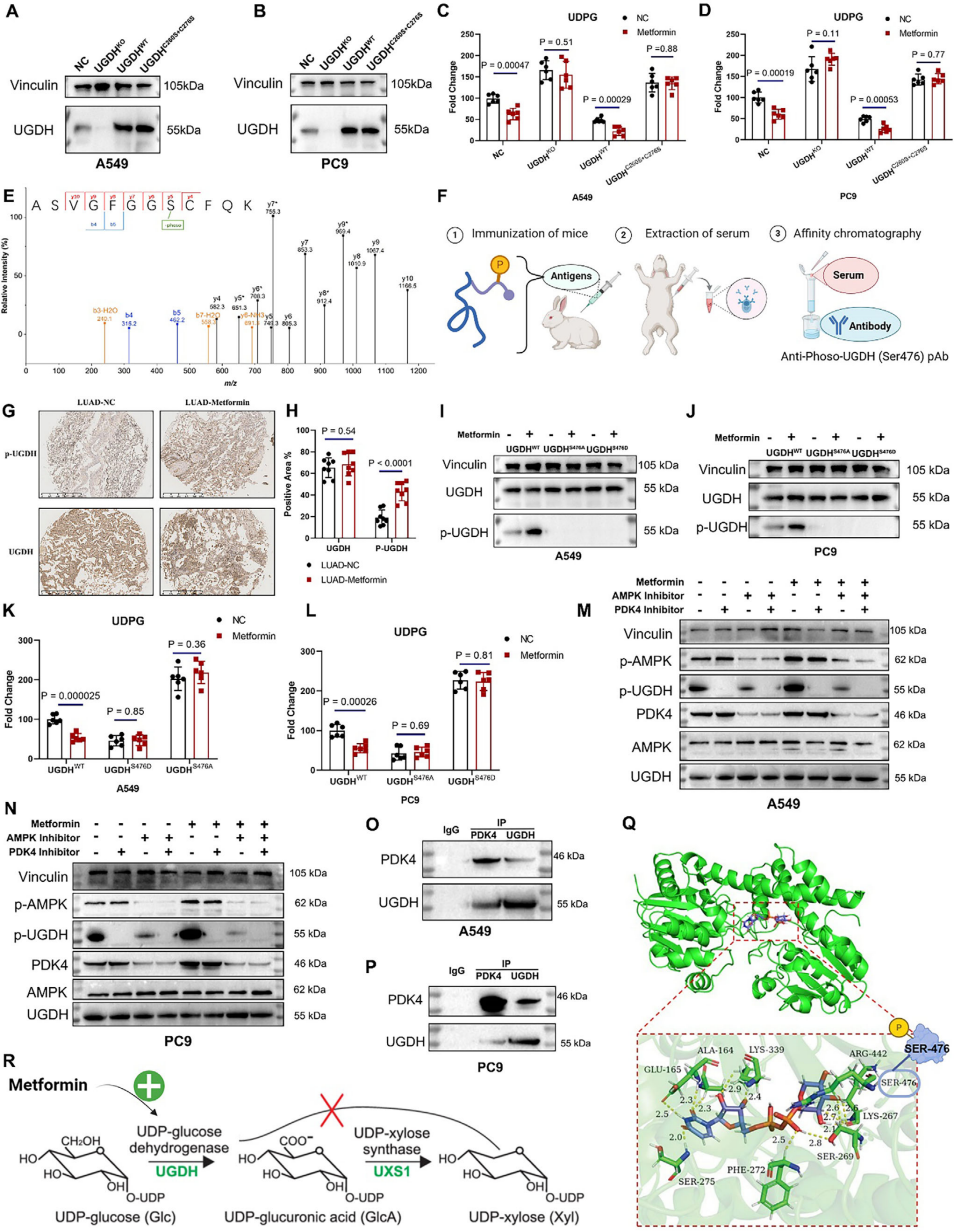
Metformin regulates UGDH phosphorylation. (A,B) Representative Western blot images showing UGDH expression in A549 and PC9 cells across different groups: control, UGDH knockout (KO), UGDH‐KO with wild‐type UGDH overexpression UGDH^WT^, and UGDH‐KO with mutant UGDH overexpression UGDH^C260S+C276S^ (n = 6 independent experiments). (C, D) Targeted metabolic mass spectrometry quantification of UDPG levels in the aforementioned A549 and PC9 cell groups (n = 6 biologically independent samples per group). (E) Phosphoproteomics analysis demonstrating that metformin treatment significantly promotes the phosphorylation of UGDH at the S476 site (n = 3 per group). (F) Schematic illustration detailing the production of the customized p‐UGDH(S476) antibody. (G,H) Representative immunohistochemistry (IHC) images and quantitative evaluation of UGDH and p‐UGDH(S476) expression (n = 8 per group). Scale bar: 625 µm. (I,J) Western blot analysis of UGDH and p‐UGDH(S476) expression in UGDH‐KO A549 and PC9 cells re‐expressing UGDH^WT^, the phospho‐dead mutant UGDH^S476A^, or the phospho‐mimetic mutant UGDH^S476D^, cultured with or without metformin (n = 3 independent experiments). (K, L) Targeted metabolic mass spectrometry evaluating the effect of metformin on intracellular UDPG levels in UGDH‐KO cells re‐expressing UGDH^WT^, UGDH^S476A^, or UGDH^S476D^ (n = 3 biologically independent samples per group). (M,N) Western blot analysis demonstrating that pharmacological inhibition of PDK4 using the specific inhibitor PDK4‐IN‐1 effectively abrogates the phosphorylation of UGDH at S476 (n = 3 independent experiments). (O,P) Co‐immunoprecipitation (Co‐IP) assays revealing a direct physical interaction between the PDK4 and UGDH proteins (n = 3 independent experiments). (Q) Molecular docking model illustrating the binding interaction between UDP‐Xyl and UGDH. (R) Schematic diagram proposing the mechanism by which metformin and UDP‐Xyl regulate UGDH activity. Statistical information: Statistical significance was determined using one‐way or two‐way ANOVA followed by Bonferroni's post hoc test for multiple comparisons (panels C, D, K, L), and the two‐tailed unpaired Student's *t*‐test for comparisons between two groups (panels E, G, H).

UGDH serves as the key enzyme catalyzing the metabolism of UDPG into UDPGA, and its structure is shown in Figure S2A. Therefore, we hypothesized whether metformin exerts its influence on glucuronic acid metabolism via targeting UGDH. By knocking out UGDH in LUAD cells, we observed that metformin's effect on UDPG levels was abolished (Figure 2A–D). Upon re‐expression of wild‐type UGDH (UGDH^WT^) following knockout, this effect was restored, indicating that metformin impacts glucuronic acid metabolism via UGDH (Figure 2A–D). Furthermore, when the mutant UGDH with deficient activity (UGDH^C260S+C276S^) was re‐overexpressed in knockout cells, metformin's effect on UDPG levels remained inhibited, further supporting that metformin acts through the enzymatic activity of UGDH rather than through its other roles, such as the protein interactions of UGDH reported in various studies (Figure 2A–D).

We subsequently investigated whether metformin altered the expression level of UGDH. However, transcriptome sequencing of LUAD cells revealed no significant changes in the expression levels of UGDH or other enzymes involved in the glucuronic acid pathway, which was also validated by qRT‐PCR (Figure S2B,C).

Could it be that metformin regulates UGDH through post‐translational modifications? Our phosphorylomics results revealed that the phosphorylation of the serine residue at position 476 (S476) of UGDH was significantly increased following metformin treatment compared to the control group (Figure 2E). An antibody specific to phosphorylated UGDH at serine 476 [p‐UGDH(S476)] was customized, and the metformin‐induced p‐UGDH(S476) was confirmed by both immunohistochemistry (IHC) and western blot (Figure 2F–H).

A comprehensive analysis of phosphorylation data from a public database has revealed that p‐UGDH(S476) is widespread in human cells [21]. Analysis of LUAD phosphoproteomics data from the CPTAC database—comprising a robust cohort of 110 tumor samples and 104 normal samples—confirmed that the phosphorylation level of UGDH S476 is significantly upregulated in LUAD tumor tissues compared to normal tissues (*p*< 2e‐16) (Figure S2D). Using the optimal cut‐off approach, with distinct thresholds applied for OS and PFS analyses, we observed a trend indicating that patients with lower UGDH S476 phosphorylation levels exhibited better Overall Survival (OS) and Progression‐Free Survival (PFS) than those with high phosphorylation levels. However, the differences did not reach statistical significance (OS p = 0.11; PFS p = 0.17, Figure S2E,F).

Subsequently, we sought to corroborate this trend using an in‐house, tightly controlled follow‐up cohort of 60 LUAD patients (with surgical specimens collected in 2022) (Figure S2G). Consistent with the CPTAC data, patients with lower p‐UGDH(S476) levels exhibited a similar trend of better OS. However, likely due to the limited sample size of this available cohort, the difference remained at borderline significance (Log‐rank p = 0.0917). These results indicate that while p‐UGDH(S476) is highly prevalent in tumors, its prognostic value in currently available datasets lacks strong statistical significance. This finding, combined with the technical challenges of robust clinical detection, underscores that its immediate clinical translation is limited and remains a subject for future investigation.

To investigate the specific effects of p‐UGDH(S476), we re‐expressed UGDH^S476D^, mimicking the phosphorylated state, and UGDH^S476A^, mimicking the dephosphorylated state, in UGDH knockout cells. The results showed that UGDH^S476D^ significantly reduced intracellular UDPG levels and enhanced the toxicity of high‐concentration UDPGA, while UGDH^S476A^ exhibited the opposite effects (Figure S2H,I). This strongly suggests that p‐UGDH(S476) significantly promotes the catalytic activity of UGDH. Further research revealed that re‐expression of either UGDH^S476D^ or UGDH^S476A^ in UGDH knockout cells completely blocked the effects of metformin on UGDH levels and its associated toxicity (Figure 2H,I).

To systematically evaluate the impact of this phosphorylation on cell phenotypes, we performed CCK‐8 cell proliferation assays using A549 and PC9 cell lines. The results demonstrated a clear growth hierarchy among cell types: under normal conditions, cells exhibited varying proliferation rates; when treated with metformin, the proliferation of WT and S476D—expressing cells was reduced to a comparable and relatively higher level, while that of S476A—expressing cells was suppressed to a much lower degree compared to the other two (Figure S2J,K).

Consistently, targeted metabolomics analyses demonstrated that the intracellular levels of UDPGA and UDP‐Xyl in cells expressing the S476A and S476D mutants exhibited no significant changes regardless of metformin treatment (Figure S2L). This lack of metabolic fluctuation firmly establishes that the metformin‐driven alteration in glucuronic acid metabolic flux relies entirely on the dynamic phosphorylation of S476.

Furthermore, we compared the tumorigenic ability of UGDH WT and S476D in nude mice. The results demonstrated that, despite sustained S476D phosphorylation (which mimics constitutive activation), tumors did not regress due to the presence of UXS1, which detoxifies UDPGA accumulation. However, metformin treatment led to a slight reduction in tumor size, suggesting that its anti‐tumor effects may involve additional pathways beyond UGDH phosphorylation alone (Figure S2M). This finding indicates that metformin promotes glucuronic acid metabolism by enhancing p‐UGDH(S476).

Given that metformin primarily activates AMPK, which in turn phosphorylates multiple downstream factors, we further investigated the role of AMPK in p‐UGDH(S476). The results showed that the addition of an AMPK inhibitor (Compound C) significantly inhibited the promotional effect of metformin on p‐UGDH(S476), strongly supporting our hypothesis that metformin promotes glucuronic acid metabolism through AMPK‐mediated p‐UGDH(S476).

However, because AMPK does not typically interact directly with UGDH, we sought to identify the intermediate kinase responsible for this specific phosphorylation event. We screened the top 20 candidate kinases predicted to phosphorylate UGDH and identified Pyruvate Dehydrogenase Kinase 4 (PDK4) as the sole candidate whose expression is actively regulated by AMPK. To validate whether PDK4 functionally targets UGDH, we treated cells with a specific PDK4 inhibitor, PDK4‐IN‐1. Western blot analysis demonstrated that pharmacological inhibition of PDK4 effectively abrogated the phosphorylation of UGDH at S476 (Figure 2M,N). To further confirm that this was a direct effect, we performed Co‐Immunoprecipitation (Co‐IP) assays, which revealed a direct physical interaction between the PDK4 and UGDH proteins (Figure 2O,P). Collectively, these results delineate a clear signaling axis wherein AMPK promotes the expression of PDK4, which subsequently binds to and directly phosphorylates UGDH at the S476 site.

Previous studies have reported that UDP‐Xyl, the downstream metabolite of the glucuronic acid pathway, allosterically inhibits the enzymatic activity of UGDH by binding to its C‐terminal region. Given that the S476 site is in close proximity to the C‐terminal region of UGDH, we hypothesize that p‐UGDH(S476) may enhance UGDH activity by impeding the binding of UDP‐Xyl to UGDH and the subsequent allosteric inhibition. The molecular docking results indicate that the binding energy between UDP‐Xyl and UGDH is significantly reduced following S476 phosphorylation (Figure 2Q; Figure S2N). The results presented further support this hypothesis (Figure 2R)

### 2.3 Cells Treated With Metformin are More Reliant on UXS1 for Detoxifying UDPGA

**FIGURE 3:**
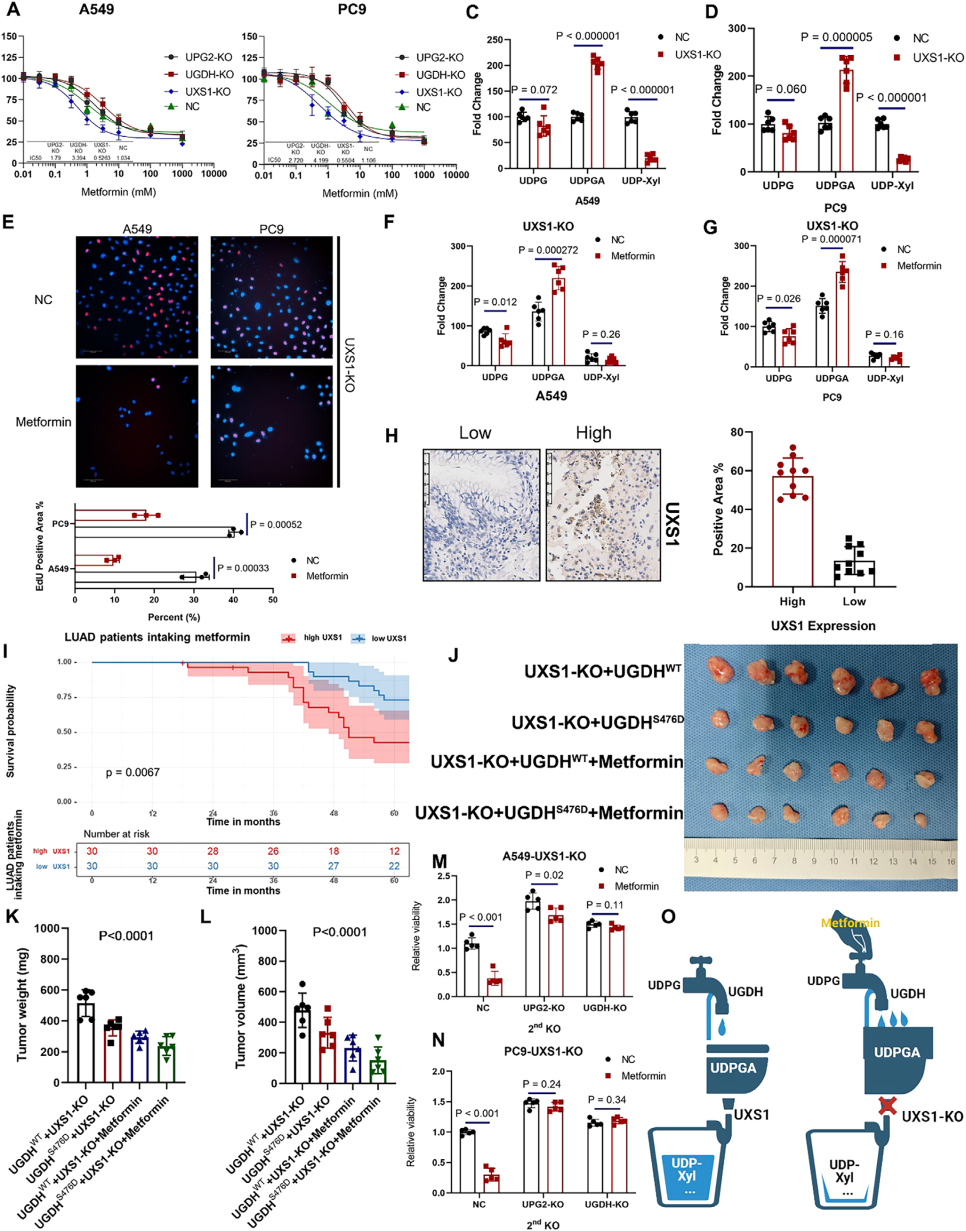
Role of metabolic enzymes in the glucuronide pathway. (A,B) CCK‐8 assays determining the half‐maximal inhibitory concentration (IC50) of metformin in A549 and PC9 cells following the knockout (KO) of UGP2, UGDH, and UXS1, respectively (n = 3 independent experiments). (C,D) Targeted metabolic mass spectrometry quantification demonstrating significantly higher UDPGA and lower UDP‐Xyl levels in UXS1‐KO A549 and PC9 cells compared to the control group (n = 6 biologically independent samples per group). (E) EdU proliferation assays validating the inhibitory effect of metformin treatment on the proliferation of UXS1‐KO A549 and PC9 cells (n = 3 independent experiments). (F,G) Targeted metabolic mass spectrometry analysis revealing a significant decrease in UDPG, a marked increase in UDPGA, and critically low levels of UDP‐Xyl in UXS1‐KO A549 and PC9 cells following metformin addition (n = 6 biologically independent samples per group). (H) Representative immunohistochemistry (IHC) images illustrating the differential expression patterns (high vs. low) of UXS1 in tumor tissues from LUAD patients (n = 10 patients). Scale bar: 625 µm. (I) Kaplan–Meier (K‐M) survival analysis demonstrating a poorer prognosis for patients with high UXS1 expression within a cohort of LUAD patients receiving metformin treatment (n = 60 patients). (J) Representative images of subcutaneous xenograft tumors derived from cells with UXS1 knockout and UGDH^S476D^ mutation, treated with or without metformin (n = 6 mice per group). (K, L) Quantitative analysis of tumor weight (K) and tumor volume (L) from the subcutaneous xenograft models described in panel J. (M,N) CCK‐8 cell viability assays indicating that subsequent secondary knockout of UGP2 or UGDH in UXS1‐KO A549 and PC9 cells abolishes the significant effect of metformin on cell viability (n = 3 independent experiments). (O) Schematic representation of the proposed “Pool model” elucidating the dynamic interplay between metformin treatment and the UGDH/UXS1 metabolic axis. Statistical information: Statistical significance was evaluated using the two‐tailed unpaired Student's *t*‐test for two‐group comparisons (panels C, D, K, L), and one‐way or two‐way ANOVA followed by Bonferroni's post hoc test for multiple group comparisons (panels A, B, E, F, G, M, N). The survival difference in panel I was assessed using the Log‐rank (Mantel‐Cox) test.

Based on the “kitchen‐sink” model, UDPGA catalyzed by metformin‐activated UGDH may make cells more reliant on UXS1 for detoxification. To test this hypothesis, we generated UGP2‐KO, UGDH‐KO, and UXS1‐KO cells in both A549 and PC9 cell lines, and assessed the effects of metformin in these cells. The results showed a significant decrease in the IC50 value of metformin in UXS1‐KO cell lines, indicating that UXS1 knockout significantly enhances the inhibition of metformin on tumor cells as well as the inhibition of production of UDP‐Xyl and the consumption of UDPGA (Figure 3A–D; Figure S3A,B). Further EdU experiments yielded similar results, demonstrating that the proliferative capacity of UXS1‐KO LUAD cells is significantly inhibited by metformin (Figure 3E; Figure S3C,D). Subsequently, using metabolic mass spectrometry, we found that UXS1 knockout, in combination with metformin, significantly increased UDPGA levels, while the content of UDP‐Xyl was significantly diminished (Figure 3F,G).

In our cohort of 60 LUAD patients taking metformin, patients were divided into two groups based on UXS1 expression levels detected by IHC. The results revealed that patients with low UXS1 expression had a better prognosis (Figure 3H,I), further confirming that tumors treated with metformin are more dependent on UXS1. Additionally, analysis of LUAD patient data from the TCGA and GEO databases showed that patients with high‐UGDH/low‐UXS1 expression had a poorer prognosis, with the result of PPI also showing a connection between UGDH and UXS1, suggesting that tumors with higher UGDH activity are more reliant on UXS1 (Figure S3E–G). To further dissect the role of UXS1, we knocked out UXS1 in lung adenocarcinoma cells and performed subcutaneous xenograft experiments. The results showed that UXS1 knockout alone led to a modest reduction in tumor size. More importantly, when UXS1 was knocked out in combination with the S476D mutation, there was a significant and marked reduction in tumor size. Notably, this effect was independent of metformin treatment, supporting the conclusion that metformin's anti‐tumor mechanism involves promoting UGDH phosphorylation, leading to UDPGA accumulation. The absence of UXS1 prevents UDPGA detoxification, resulting in cytotoxic effects that inhibit tumor growth (Figure 3J–L).

We then used lung adenocarcinoma cells in which UXS1 was knocked out (A549‐UXS1‐KO, PC9‐UXS1‐KO). By employing cell viability to compare the second knockout of UGP2 and UGDH in A549‐UXS1‐KO and PC9‐UXS1‐KO cells, we discovered that following the addition of metformin, a significant decrease in cell viability was observed exclusively in A549 and PC9 cells with UXS1 knockout alone. Conversely, no substantial impact on cell viability was observed when metformin was administered in A549‐UXS1‐KO, PC9‐UXS1‐KO cells with secondary UGP2 or UGDH knockout (Figure 3M,N; Figure S3H,I). Our resulting pool model, designated UXS1, functions as a “drainpipe” that facilitates the conversion of toxic UDPGA to non‐toxic UDP‐Xyl in tumor cells with high expression of UGDH. Metformin, in turn, modulates the post‐translational modification of UGDH, thereby further enhancing the production of UDPGA (Figure 3O).

### 2.4 Identification of Compounds Specifically Targeting UXS1 and Synergistically Sensitizing Metformin

Our findings indicate that targeting UXS1, highly expressed in various types of tumors, particularly those derived from lung cancer (Figure S4A), the pivotal enzyme involved in UDPGA detoxification, exhibits a pronounced enhanced inhibitory effect when combined with metformin. However, a compound specifically targeting UXS1 is currently unreported. Therefore, we endeavored to identify and evaluate small‐molecule inhibitors that can specifically target UXS1 through virtual screening of compound libraries, with the objective of enhancing the antitumor efficacy of metformin.

**FIGURE 4:**
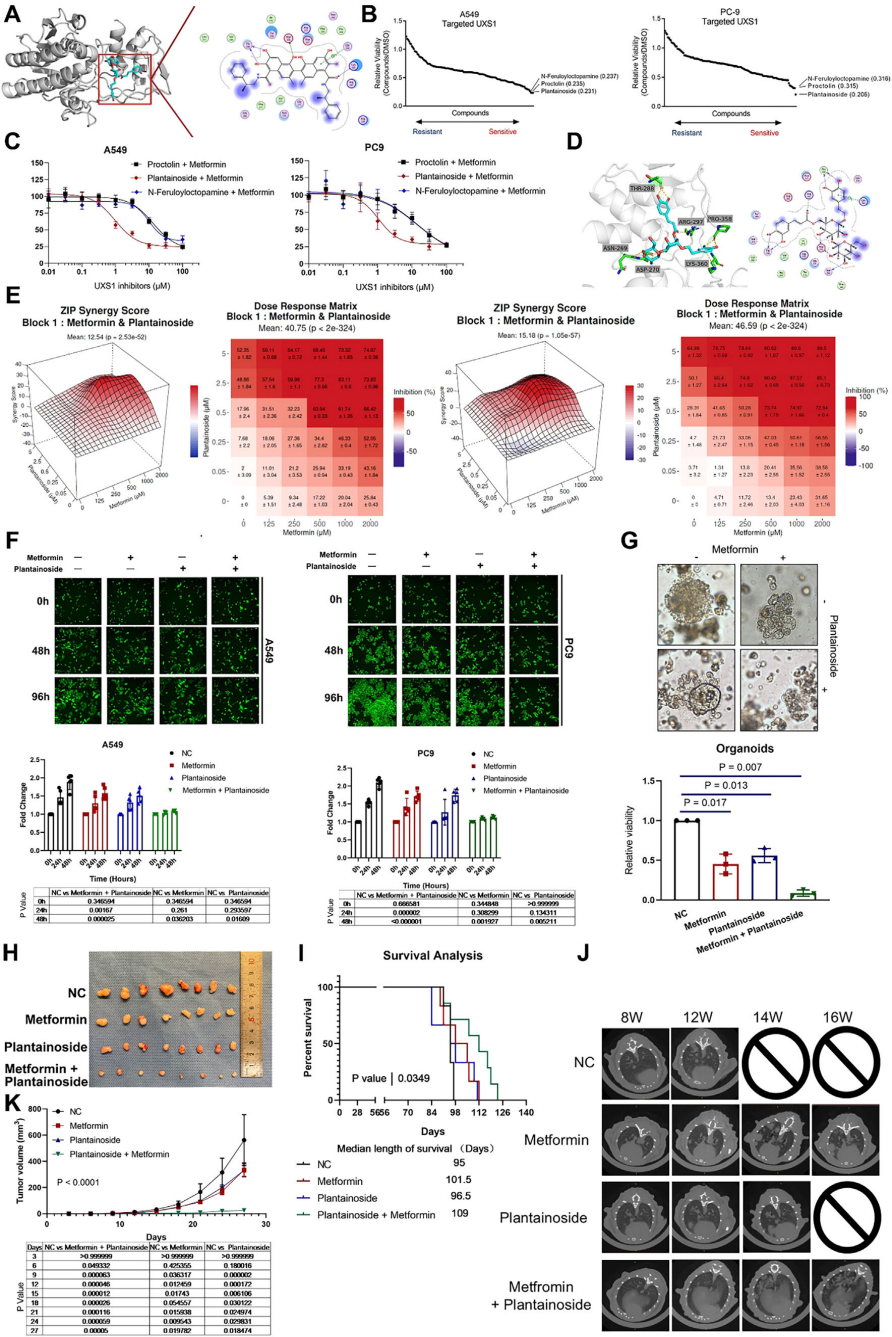
Targeting UXS1 combined with metformin causes synergistic lethality. (A) 3D structural modeling of UXS1 and schematic representation of the virtual screening process for small‐molecule targeting compounds. (B) Evaluation of the sensitizing effect of metformin in A549 and PC9 cells treated with 10 µM of the identified candidate small molecules compared to the DMSO vehicle control (n = 3 independent experiments). (C) CCK‐8 assays comparing the half‐maximal inhibitory concentration (IC_50_) in A549 and PC9 cells treated with three distinct UXS1 inhibitors in combination with 1 mM metformin (n = 3 independent experiments). (D) 2D and 3D chemical structures of the selected lead compound, plantainoside. (E) Evaluation of the synergistic inhibitory effect of metformin combined with plantainoside in A549 and PC9 cells using the ZIP regression model. The model, which integrates the Bliss and Loewe reference models, confirms the significant synergistic effect of the combination therapy (n = 3 independent experiments). (F) Cell proliferation assays evaluating the combination effect of plantainoside and 1 mM metformin vs. either plantainoside or metformin alone in A549 and PC9 cells (n = 3 independent experiments). (G, H) Representative images and quantitative analysis of the tumor‐suppressive effects in a lung cancer organoid model (G) (n = 3 organoids per group) and a Cell Line‐Derived Xenograft (CDX) nude mouse model (H) (n = 6 mice per group), demonstrating the strongest antitumor efficacy in the plantainoside and metformin combination group. (I) Tumor growth curves monitoring the progression of the CDX model over time (n = 6 mice per group). (J) Quantitative analysis of periodic CT imaging reviews, verifying the significantly slower tumor growth rate in the spontaneous lung cancer model treated with the combination of plantainoside and metformin (n = 6 mice per group). (K) Kaplan–Meier survival analysis of the spontaneous lung cancer mouse model, showing that the plantainoside and metformin combination group exhibited the longest overall survival time (n = 6 mice per group). Statistical information: Statistical significance was determined using one‐way or two‐way ANOVA followed by Bonferroni's post hoc test for multiple group comparisons (panels B, C, E, F, G, H, I, J). The survival difference in panel K was assessed using the Log‐rank (Mantel‐Cox) test.

Initially, we conducted an in‐depth analysis of the binding mode between UXS1 and its substrate UDPGA using MOE‐Site Finder software, and successfully located the inhibitor binding pocket on UXS1 (Figure 4A; Figure S4B,C). Subsequently, we performed a comprehensive virtual screening of a compound library containing 17,676 small molecules (detailed screening parameters and filtering criteria are provided in the Materials and Methods section), and identified 126 compounds with potential binding to UXS1. Through high‐content screening, we further confirmed the synergistic lethal effect of these compounds at a concentration of 10 µM when combined with metformin, and ultimately identified plantainoside as the compound that can most effectively target UXS1 and exhibits the optimal synergistic lethal effect with metformin (Figure 4B).

To functionally validate that plantainoside specifically targets UXS1 and its associated metabolic flux, we performed a key genetic rescue experiment. We generated UGDH‐knockout (UGDH‐KO) cells in both A549 and PC9 backgrounds, essentially turning off the upstream “faucet” for UDPGA production. Strikingly, cell viability assays revealed that while plantainoside significantly induced cytotoxicity in wild‐type cells, the UGDH‐KO cells were almost completely resistant to its effects (Figure S4D). This result provides compelling genetic evidence that the toxicity of plantainoside is strictly dependent on the flux through the hexosamine biosynthesis pathway culminating at UXS1. When UDPGA production is halted by UGDH knockout, the substrate pool for UXS1 is depleted, thereby rescuing the cells from the toxicity of UXS1 inhibition. This perfectly aligns with our “faucet‐off” model and offers robust biochemical and genetic validation that plantainoside's primary mechanism of action is mediated through inhibiting UXS1 activity.

We conducted CCK8 and EdU experiments, and the results consistently demonstrated that the combination of plantainoside and metformin significantly enhanced the cytotoxicity toward tumor cells (Figure 4C,D; Figure S4E). The ZIP regression model, which integrates the Bliss and Loewe models with the assumption of no interaction effect, was employed to assess the synergistic inhibitory effect of metformin and plantainoside. The results once again confirmed the significant synergistic effect of their combination (Figure 4E).

In vitro experiments revealed that the combination of metformin and plantainoside markedly inhibited the proliferation, migration, and invasion of LUAD cells (Figure 4F; Figure S4E,F). We further established organoid models and subcutaneous xenograft models in nude mice. The experimental results showed that the combination exhibited significant and synergistic inhibitory effects on both organoid and subcutaneous tumor growth in nude mice (Figure 4G–I; Figure S4G,H).

Lastly, we conducted long‐term observations of mice with spontaneously formed tumors using CT imaging. The results indicated that the mice in the combination group had the longest survival time, and their tumor growth rate was significantly slower than that in the metformin or plantainoside monotherapeutic groups (Figure 4J,K), further confirming the synergistic lethal effect of the combination of metformin and plantainoside.

### 2.5 Targeting UXS1 in Combination With Metformin Synergistically Activates Tumor Immunity and Enhances Immunotherapy Efficacy

Previous studies have demonstrated that UDPGA is closely associated with tumor immune evasion, one of the cancer hallmarks. Consequently, we further investigated the impact of targeting UXS1 in combination with metformin on tumor immunity. By analyzing the single‐cell sequencing data of tumor tissues from lung adenocarcinoma patients that we previously published [22], we found that UXS1 is predominantly expressed in tumor cells, with low expression across various immune cell types (Figure S5A,B), suggesting that targeting UXS1 does not directly affect immune cells.

**FIGURE 5:**
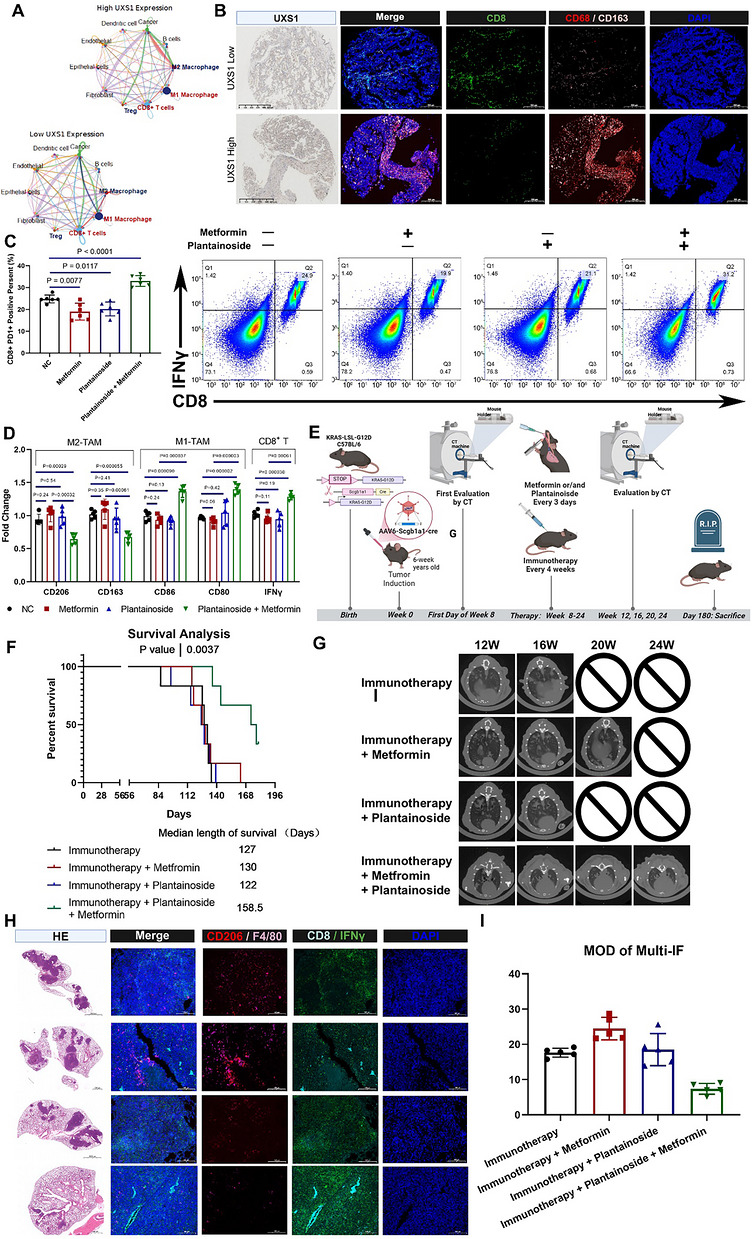
Effect of targeting UXS1 in combination with metformin on the immune microenvironment of LUAD. (A) Differences in the association of tumor cells with immune cells between high and low UXS1 expression groups in patients with LUAD taking metformin (n = 15 patients per group). (B) Representative immunohistochemistry (IHC) and immunofluorescence (IF) images showing the infiltration of T‐cells (CD8) and M2‐type tumor‐associated macrophages (M2‐TAM: CD68, CD163) in tumor tissues of LUAD patients taking metformin, stratified by high and low UXS1 expression (n = 6 patients per group). Scale bars: 625 µm for IHC and 500 µm for IF. (C,D) Flow cytometry (C) and qRT‐PCR (D) analyses comparing the expression of CD8 and IFNγ in T cells, as well as macrophage differentiation markers (M1‐TAM: CD86, CD68; M2‐TAM: CD206, CD163), following co‐culture with LUAD cells. The LUAD cells were pre‐treated with control vehicle, metformin alone, plantainoside alone, or the combination of metformin and plantainoside (n = 3 independent experiments). (E) Flowchart illustrating the experimental design of the spontaneous lung cancer mouse model. The study includes four treatment groups: immunotherapy alone, immunotherapy + metformin, immunotherapy + plantainoside, and immunotherapy + metformin + plantainoside (n = 6 mice per group), detailing model establishment, treatment timeline, and sample collection. (F) Kaplan–Meier survival curves comparing the overall survival time across the four aforementioned treatment groups (n = 6 mice per group). (G) Quantitative analysis of serial computed tomography (CT) scans assessing tumor growth kinetics in the four treatment groups (n = 6 mice per group). (H,I) Representative histological (H&E) and immunofluorescence staining of mouse lung tissues from the four treatment groups, evaluating the infiltration of M2‐TAMs and CD8^+^ IFNγ^+^ T cells (n = 3 biologically independent samples per group). Scale bars: 1000 µm for HE and 500 µm for IF. Statistical information: Statistical significance was determined using the two‐tailed unpaired Student's *t*‐test for comparisons between two groups (panels A, B), and one‐way or two‐way ANOVA followed by Bonferroni's post hoc test for multiple group comparisons (panels C, D, G, H, I). The survival differences in panel F were evaluated using the Log‐rank (Mantel‐Cox) test.

Based on UXS1 expression levels, we stratified tumor cells in the single‐cell data into high and low expression groups and examined the associations between tumor cells and immune cells within each group. Our results indicated that tumor cells with high UXS1 expression are closely linked with M2 macrophages, Tregs, and fibroblasts, whereas those with low UXS1 expression are tightly associated with M1 macrophages, CD8+ T cells, and fibroblasts (Figure 5A). Additionally, we employed the CIBERSORT algorithm to assess the relative infiltration levels of various immune cells in the tumor microenvironment (TME) of lung adenocarcinoma patients from the TCGA database. The results revealed that the immune environment in the high UXS1 expression group is more suppressive, characterized by decreased CD8+ T cell scores, reduced M1 macrophages, and increased M2 macrophages (Figure S5C).

Therefore, we focused on exploring the interplay between UXS1 and CD8+ T cells, as well as macrophages. Using multiplex immunofluorescence techniques, we evaluated the expression of CD8 and markers for M2 macrophages (CD68 and CD163) in tissues from lung adenocarcinoma patients treated with metformin. The findings showed that the low UXS1 expression in tumor cells was associated with higher infiltration of CD8+ T cells and lower M2 macrophages, contrasting sharply with the high UXS1 group (Figure 5B).

Furthermore, we established a co‐culture system of tumor cells with T cells and macrophages (Figure S5D) and discovered a synergistic effect when metformin was combined with plantainoside in LUAD cells co‐cultured with T cells and M0 macrophages. After 48 h of co‐culture, compared to the metformin‐monotherapy group, CD8 expression in T cells was significantly increased after the treatment of metformin combined with plantainoside. This group also showed a notable decrease in M2 cells and a significant increase in M1 cells (Figure 5D). We also utilized flow cytometry to quantitatively detect the expression of CD8 and IFNγ in T cells under co‐culture conditions. Based on our sequential gating strategy, we sequentially gated for lymphocytes (FSC‐A vs. SSC‐A), single cells (FSC‐H vs. FSC‐A), total CD3+ T cells, and CD8+ T cells, before finally quantifying the target CD8+ IFN‐γ+ effector populations. The quantitative results demonstrated that the proportion of CD8+ IFNγ+ double‐positive effector T cells was significantly highest in the combination group. Crucially, comprehensive pairwise statistical comparisons across all four groups confirmed that the combination treatment significantly outperformed both the control and each single‐agent group, validating a robust synergistic activation of T cells in vitro (Figure 5C).

Finally, we established a spontaneous lung cancer model via AAV‐induced KRAS activation and regularly monitored the size of lung tumors using CT scans (Figure 5E). The results demonstrated that mice in the group receiving immunotherapy combined with metformin and plantainoside exhibited the most significant tumor regression. Regarding the overall survival (Figure 5F,G), while the limited sample size (n = 6 per group) and strict ethical endpoints (mandatory euthanasia due to maximum tumor burden) precluded the achievement of statistical significance in adjusted pairwise comparisons between the combination and single‐agent groups, the overall Log‐rank test indicated a general difference across the cohorts (Overall p = 0.0037). More importantly, the combination therapy conferred the longest median survival time compared to the vehicle and monotherapy groups, providing a substantially enhanced survival benefit. Further histological and immunofluorescence staining analysis of the mouse lungs revealed that the immunotherapy plus metformin and plantainoside group had a marked reduction in M2‐TAM infiltration within the tumors and a significant increase in the proportion of CD8+ IFNγ+ T cells (Figure 5H). These findings collectively suggest that plantainoside and metformin exhibit synergistic immune‐activating effects in vitro and provide an enhanced therapeutic efficacy in vivo.

## 3 Discussion

While the majority of laboratory studies predominantly spotlighted the diverse anti‐cancer effects of metformin, its anti‐tumor efficacy has unfortunately eluded validation in several randomized controlled trials [10, 13, 23, 24]. The role of metformin in tumors is an enigmatic puzzle that remains incompletely deciphered, largely due to its wide range of targets and effects, particularly within the realm of tumor metabolism [25, 26, 27]. In this study, we unveil a hitherto unreported and highly novel discovery that metformin promotes glucuronic acid metabolism and the production of UDPGA, which showcases a paradoxical dual role in tumors, fostering growth at low concentrations while inflicting toxic effects at high concentrations, thus adding a new layer of complexity to the already intricate role of metformin in cancer.

As one of the primary targets of metformin, AMPK exhibits both tumor‐promoting and tumor‐suppressing effects [28], while UGDH has been reported as a significant tumor target across various types of cancer [17, 29, 30, 31, 32]. In this study, utilizing phosphoproteomics and a customized p‐UGDH(S476) antibody, along with the inhibitor of the AMPK pathway, we discovered that metformin phosphorylates the S476 site of UGDH through the activation of AMPK. Although phosphorylation at the S476 site of UGDH was present in previous phosphoproteomic data from cancer patients, it went unnoticed [21, 33]. We are the pioneers in conducting an in‐depth investigation into the specific function of this phosphorylation, and the first to demonstrate that this phosphorylation relieves the allosteric inhibition of UGDH by UDP‐Xyl. It is particularly noteworthy that AMPK, as an initiator of numerous intracellular phosphorylation cascades and the primary target of metformin, probably does not directly phosphorylate UGDH; instead, a downstream kinase of AMPK probably takes over, which deserves in‐depth exploration in the future [28, 34, 35]. Furthermore, UGDH harbors other post‐translational modifications worthy of in‐depth investigation [36, 37, 38]. For instance, previous studies have reported that phosphorylation of UGDH at tyrosine 473, induced by EGFR activation, interacts with Hu antigen R (HuR) and enhances the stability of SNAI1 mRNA, thereby promoting the progression of lung cancer [38, 39]. UGDH lactylation at K6 in chondrocytes caused by lactate impairs its function, disrupts glycosaminoglycan synthesis and nuclear‐cytoplasmic distribution, and contributes to OA development through MAPK pathway activation [36].

In the study by Doshi et al. [19], it was revealed that tumor cells producing high levels of UDPGA rely on UXS1 to detoxify the toxicity associated with these elevated concentrations, a mechanism termed the “kitchen‐sink” model [19]. Building on this finding, we trustworthily demonstrated that knocking out UXS1 exerts a remarkable synergistic effect with metformin, leading to the accumulation of exceptionally high levels of UDPGA in tumor cells and ultimately resulting in synthetic lethality. Our results also suggest that it may be feasible to identify tumor patients with heightened sensitivity to metformin by scrutinizing their UXS1 expression levels. Moreover, we employed virtual screening technology to meticulously sift through thousands of small‐molecule compounds and pinpointed plantainoside as a highly specific inhibitor of UXS1. Rigorous experimental validation has unequivocally confirmed the immense therapeutic potential of combining plantainoside with metformin, or even enhancing this combination with immunotherapy, as a new strategy for the treatment of cancer.

In this study, we generated patient‐derived organoids (PDOs) from various lung adenocarcinoma patients and convincingly demonstrated that the combination of plantainoside and metformin synergistically inhibited organoid growth. Notably, patient‐derived organoids offer an unparalleled platform for drug sensitivity testing, as they meticulously retain the histological and molecular features of the original tissues [40, 41, 42]. Meanwhile, in addition to nude mice xenograft models, we also utilized mice harboring the Kras G12D mutation and expressing humanized UGDH to establish spontaneous lung cancer models, which provide a far more accurate representation of the immune microenvironment compared to nude mice xenografts. Our remarkable findings in these spontaneous lung cancer models underscore the untapped therapeutic potential of combining plantainoside with metformin and suggest the further potential for combining this treatment with immunotherapy.

### 3.1 Limitations of the Study

Several limitations of this study must be acknowledged, particularly regarding the immediate translational and prognostic value of our findings. While our analysis of CPTAC phosphoproteomics data demonstrates that p‐UGDH(S476) is significantly upregulated in lung adenocarcinoma (LUAD) tissues compared to normal tissues, its definitive prognostic relevance requires further validation. Furthermore, the robust clinical quantification of p‐UGDH(S476) in patient tissues currently presents significant technical challenges. Translating these findings into clinical practice will necessitate the development of highly reliable detection methods, such as targeted phospho‐mass spectrometry (phospho‐MS) or rigorously validated phospho‐specific antibodies that incorporate strict specificity controls (e.g., λ‐phosphatase treatment, peptide competition, or S476A‐mutant loss‐of‐signal verification). Until such standardized quantification protocols are established and evaluated in larger, independent cohorts, the clinical utility of p‐UGDH(S476) remains a subject for future investigation.

## 4 Methods

### 4.1 Ethics Approval Statement

All methods were carried out in accordance with relevant guidelines and regulations. All participants of the clinical study signed informed consent according to the ethical requirements in the Declaration of Helsinki, which was approved by the Ethics Committee of Zhongshan Hospital, Fudan University (B2021–267). Written informed consent to participate and to publish was obtained from participants before inclusion in the study.

All animal experiments complied with the WMA Statement on animal use in biomedical research and EU recommendations (Directive 2010/63/EU) for experimental design and analysis in pharmacology care, and animal experiments were approved by the Ethics Committee of Zhongshan Hospital, Fudan University (2022–203).

### 4.2 Cell Culture

The human lung adenocarcinoma cell line A549, PC9, and the human monocytic leukemia cell line THP1 were procured from the American Type Culture Collection (Manassas, VA, USA) and maintained in the laboratory of the Thoracic Surgery Laboratory of Zhongshan Hospital, Fudan University. The cells were authenticated by STR profiling every 3 months. All cell lines tested negative for mycoplasma contamination. The A549 cells were cultured in Dulbecco's Modified Eagle's Medium (DMEM, Gibco‐Life Technologies) supplemented with 10% fetal bovine serum (FBS, Every Green, Zhejiang, China) and 100 µg/mL of Penicillin‐Streptomycin‐Gentamicin Solution (Beyotime, China).

THP1 was cultured in RPMI‐1640 (Gibco‐Life Technologies) supplemented with 10% fetal bovine serum (FBS, Gibco, New Zealand) and 100 µg/mL of Penicillin‐Streptomycin‐Gentamicin Solution (Beyotime, China). The cells were incubated in a humid atmosphere containing 5% CO_2_ at 37°C.

PBMC (peripheral blood mononuclear cells) in human blood were separated using a lymphocyte isolation solution, and CD8+ T cells were subsequently sorted from these using a CD8+ T cell isolation kit (Item No. 130‐096‐495) from Metheni. Anti‐CD3/CD28 magnetic beads (Thermofisher, item no. 1131D) were then added and incubated for 2 h to activate the above CD8+ T cells. Subsequent to this, the cells were co‐cultured with a range of tumour cells. The expression of CD8, IFNγ, and TNFα was then analyzed via flow cytometry, while ELISA was used to detect IFNγ and TNFα in the cell culture supernatants.

The OT‐1 mice (C57BL/6‐Tg (TcraTcrb)1100Mjb/Crl) were procured from Golden Pharmaceuticals (Nanjing, China) and were housed in conditions free of specific pathogens, with a 12 h light‐12 h dark cycle. Mice were euthanized at 6–8 weeks of age, and spleens were collected and minced with the flattened end of a piston. The released splenocytes were filtered and suspended in erythrocyte lysis buffer (Beyotime) for 2 min. Thereafter, the splenocytes were incubated in RPMI 1640 medium (Aladdin, China) containing 2.5 µg/mL OVA257‐264 peptide (MedChemExpress), 10 ng/mL mouse recombinant IL‐2 (MedChemExpress), and 40 µM 2‐mercaptoethanol (Aladdin, China). The cells were then cultivated in RPMI 1640 medium (Hyclone, USA) at 37°C for 4 days. The expanded OT‐1 T cells were subsequently purified using the MojoSort Mouse CD8 T Cell Isolation Kit (BioLegend, USA).

OVA+ Luc+ tumor cells were co‐cultured with OT‐1 T cells. Mouse LLC cells with or without metformin or plantainoside were infected with lentivirus expressing ovalbumin (OVA+) and nano luciferase (Luc+). Quantification of luciferase activity was performed to correct for differential ovalbumin expression in different cell groups.

### 4.3 Patient Samples

Samples from patients diagnosed with lung adenocarcinoma were obtained from the Department of Thoracic Surgery, Zhongshan Hospital, Fudan University, Shanghai, China. Patients were required to provide written informed consent in accordance with the ethical guidelines set forth by the Zhongshan Hospital, Fudan University. The organoid models were created in accordance with the methodology outlined in the section *Tumor organoid generation* (Table S2).

### 4.4 Online Database Analyses

The Kaplan‐Meier (KM) plotter (http://kmplot.com/analysis/) was utilized to compare the overall survival and prognosis differences. The TCGA data portal (http://cancergenome.‐nih.gov/) was employed to analyze the expression levels of UXS1 in LUAD patients.

### 4.5 LC‐MS/MS Analysis

Initially, the tryptic peptides were dissolved, and then directly loaded onto a reversed‐phase analytical column. The peptides were then separated using an Easy‐nLC1000 UHPLC system (Bruker Daltonics, Beijing, China), with a subsequent capillary source and timsTOF Pro mass spectrometry. The MS/MS data were preprocessed with the MaxQuant search engine (v1.6.15.0). The peptide length was set to a minimum of seven, and the number of modifications for each peptide was limited to five. The false discovery rate was adjusted to less than one per cent for proteins, peptides, and PSMs.

### 4.6 Single‐Cell Sequencing and Data Processing

Single‐cell RNA sequencing data were obtained from LUAD samples, as previously described [22, 43]. Following data acquisition, rigorous quality control measures were implemented. These measures were followed by dimensionality reduction and unsupervised clustering analyses to classify cell types and assess gene expression patterns.

### 4.7 Virtual Screening of Inhibitors Targeting UXS1

In this study, a comprehensive structure‐based virtual screening was performed targeting the substrate (UDP‐glucuronic acid) binding site of the human UXS1 protein. The TargetMol compound library was selected as the screening database. The 3D crystal structure of UXS1 (PDB ID: 4GLL) was pre‐processed using the Protein Preparation Wizard module in the Schrödinger software suite. This preparation step involved optimizing bond orders, adding hydrogen atoms, assigning disulfide bonds, and determining protonation states at pH 7.0 using the PROPKA method. Subsequently, a restrained energy minimization was conducted utilizing the OPLS4 force field until the root‐mean‐square deviation (RMSD) of heavy atoms converged to 0.3 Å, thereby effectively resolving interatomic steric clashes and optimizing side‐chain conformations.

Concurrently, the small‐molecule compound library was prepared using the LigPrep module. The Epik method was employed to generate protonation states and tautomers at pH 7.0 ± 2.0 while preserving the original stereochemistry, with up to 32 conformations generated per molecule to ensure global conformational coverage. The molecular docking and screening pipeline employed a step‐wise filtering strategy comprising Standard Precision (SP), Extra Precision (XP), and Molecular Mechanics/Generalized Born Surface Area (MM‐GBSA) calculations. Initially, flexible ligand docking was performed in SP mode, and the top 30% of the ranked molecules were retained for subsequent XP mode docking. The top 30% of the XP results were then subjected to rigorous MM‐GBSA calculations to estimate relative binding free energies.

To further refine the hit compounds, Protein‐Ligand Interaction Fingerprints (PLIF) analysis was conducted to evaluate key intermolecular interactions. Compounds were strictly filtered based on a dual‐threshold criterion: an MM‐GBSA score of < −40 Kcal/mol and an XP docking score of < −6 Kcal/mol, which yielded 430 candidate molecules. To ensure chemical diversity, structural clustering was performed using MOE software (2024, Chemical Computing Group ULC). Compounds exhibiting ≥ 70% structural similarity were clustered together, generating 183 distinct structural clusters. The representative molecule with the most favorable binding profile from each of the 183 clusters was initially selected. Following a final rigorous visual inspection of their 3D binding modes to eliminate false positives and ensure optimal pocket occupation, a final refined set of 126 candidate compounds was identified and advanced for subsequent in vitro high‐content screening.

### 4.8 Plasmid Construction and Validation Information

To establish gene‐edited cell models, we performed CRISPR/Cas9‐mediated knockout of three genes (UGDH, UPG2, UXS1) in A549 and PC9 cell lines. All lentiviral particles (including those for gene knockout and UGDH variant overexpression) were customized by GENECHEM (Shanghai, China).

For UGDH knockout, cells were transduced with lentiviral particles encoding Cas9 and a UGDH‐targeting sgRNA (sequence: 5’‐AAGATCTGTTGCATCGGTGC‐3’). For UPG2 knockout, a separate lentivirus carrying sgRNA (5’‐AAGAATATACAGATCCACTG‐3’) was used, while UXS1 knockout employed sgRNA (5’‐AGTTGACCAGATATACCATC‐3’). All transduced cells underwent puromycin (Beyotime) selection for 72 h, followed by single‐clone isolation via limiting dilution. Successful knockout of each gene was validated by Western blot analysis.

For re‐expression studies, UGDH‐KO cells were further transduced with lentiviral vectors overexpressing wild‐type UGDH (UGDHWT), a phosphorylation‐deficient mutant (UGDHS476A), or a phosphorylation‐mimicking mutant (UGDHS476D). Stable transfectants were selected with blasticidin (Beyotime) for 7 days, and overexpression efficiency was confirmed by Western blot analysis.

### 4.9 Lentiviral Transduction

Lentiviral solutions containing HBLV‐h‐GSDMD‐3xflag plasmid and UGDH‐KO sgRNA plasmid (harboring puromycin resistance and green fluorescent protein [GFP] fluorescence) were purchased from Shanghai Genechem Co., Ltd (Shanghai, China). After the cells reached a density of 50%, the lentiviral solution and polybrene (5 µg/mL) were mixed with half of the original complete medium volume and used to infect the cells. The complete medium was replenished after 4 h and replaced with a fresh complete medium after 24 h. GFP fluorescence intensity was evaluated after 48 h. After achieving a 60%–70% cell density, the complete medium containing the appropriate puromycin concentration was replaced to screen the cells and construct stable cell lines for subsequent experiments.

sgUGDH sequence: AAGATCTGTTGCATCGGTGC

### 4.10 Cellular Toxicity Assays

Cells were cultivated in 96‐well plates at a suitable density for an overnight period. Following the administration of treatments, cell viability was measured using the Cell Counting kit‐8 (TargetMol) in accordance with the manufacturer's protocols. The absorbances of each well were determined using a microplate reader (SpectraMax iD3, Molecular Devices, San Jose, CA, USA).

### 4.11 Cell Proliferation

The EdU Cell Proliferation Kit (E607204, Sangon Biotech) was utilized for the cell proliferation assay. A total of 2.5 × 10^4^ A549 or PC9 cells were seeded in 24‐well plates. Following a 24 h incubation period, complete adhesion of the cells to the wall was observed. Metformin or plantainoside was then administered for detection 48 h later. As previously outlined [44], a comprehensive procedural guide has been provided.

For the analysis utilizing the high‐content imaging system, as reported in previous research [45], cells were infected with lentivirus carrying the Green Fluorescent Protein (GFP) sequence and then seeded at a density of 1800 cells per well. The Operetta CLS High‐Content Analysis System (PerkinElmer, Waltham, USA) was used for capturing the fluorescence images of the cells.

### 4.12 Cell Migration and Invasion

The Transwell chamber was initially positioned within a 24‐well plate, followed by the addition of medium and inducer to the lower chamber. Subsequently, the cell suspension was introduced into the upper chamber and incubated in an incubator at 37°C for a duration of 48 h. Upon the cells migrating to the lower chamber, the Transwell device was removed, and the cells were subjected to fixation with formaldehyde, staining with methyl violet, and documentation via photography.

For the invasion experiment, 30 µg of Matrigel matrix was spread on the filter membrane in the upper chamber of the Transwell. Following a 72 h culture period, the Transwell apparatus was retrieved, and the cells were formaldehyde‐fixed, methyl violet‐stained, and imaged.

### 4.13 RNA Analysis

Total RNA extraction was performed using the TRIzol reagent (Tiangen Biotech, Beijing, China), in strict accordance with the manufacturer's guidelines. Subsequent to this procedure, the concentration and purity of the extracted RNA were determined by spectroscopic analysis using a Denovix DS‐11 spectrophotometer (Denovix, Wilmington, DE, USA). The reverse transcription‐quantitative polymerase chain reaction (RT‐qPCR) was then performed on the RNA samples, converted into cDNA using the Hifair II First‐Strand cDNA Synthesis Kit (YEASEN, Shanghai, China). RT‐qPCR was carried out utilizing the Hieff qPCR SYBR Green Master Mix (YEASEN) on an ABI QuantStudio 5 real‐time PCR system (Thermo Fisher Scientific, Waltham, MA, USA), and the results were analyzed. The quantification of the target gene expression was then conducted by utilizing the 2^‐ΔΔCT method, with the normalization to the expression level of the housekeeping gene (β‐actin) being employed as the internal control. The complete primer list that was used in this study can be found in Table S3.

### 4.14 Western Blot Analysis

The samples were loaded onto a 10% SDS‐PAGE gel, and western blotting was performed as previously described. The gels were then transferred onto Immuno‐blot polyvinylidene fluoride membranes (Merck‐Millipore, Burlington, MA, USA) and subsequently blocked. The membranes were then subjected to immunoblotting with the following antibodies: AMPKα1 (Ab37 59, diluted 1:1000, Abcam), p‐AMPKα1 (phosphor S48) (Ab131357, diluted 1:1000, Abcam), UGDH (AG3528, diluted 1:1,500, Beyotime), p‐UGDH (diluted 1:1000), Vinculin (AG3539, diluted 1:3000, Abways), overnight at 4°C. Following three washes in TBS with 0.1% Tween, the membrane was then incubated for 1 h at room temperature with an appropriate horseradish peroxidase (HRP)‐conjugated secondary antibody. The following secondary antibodies were used in this study: goat anti‐rabbit IgG (1:3000) and goat anti‐mouse IgG (1:3000). These were purchased from Beyotime (China). The bands were then detected as previously described [46, 47].

Detailed methods for generating the customized p‐UGDH(S476) antibody: We customized a phospho‐UGDH(S476)‐specific antibody in collaboration with Abclonal Technology (Wuhan, Hubei, China). The workflow started with synthesizing a phosphorylated peptide (PYAP‐(p‐S)‐GEI‐C) incorporating the target serine 476 modification, which was used for five sequential rabbit immunizations. Post‐immunization sera underwent affinity purification: positive selection with the phosphorylated peptide enriched phospho‐specific antibodies, while negative depletion against the non‐phosphorylated control (PYAPSGEI‐C) removed cross‐reactive components. Antibody specificity was validated via dot blot analysis, where the phosphorylated peptide was spotted onto PVDF membranes, blocked to prevent non‐specific binding, and detected using the purified primary antibody followed by a horseradish peroxidase (HRP)‐conjugated goat anti‐rabbit secondary antibody.

### 4.15 Enzyme‐Linked Immunosorbent Assay (ELISA)

The enzyme‐linked immunosorbent assay (ELISA) of UDPG was performed using a Human UDPG ELISA Kit (FT‐P36342R, Fantaibio). The microplate, which had been pre‐coated with Anti‐Human UDPG antibody, was then added with cell supernatant and standard. Following this, the microplate was incubated, and then biotin‐conjugated Anti‐Human UDPG antibody was added. This is then combined with HRP‐conjugated streptavidin to form an immune complex, which is then incubated and washed to remove unbound enzyme. The next step is to add this to the chromogenic substrate TMB, which produces a blue color. This is then converted to the final yellow under the action of acid. Finally, the OD value is measured at 450 nm. The concentration of UDPG in the sample can then be calculated using a standard curve.

### 4.16 Flow Cytometry

The quantification of IFNγ in T cells was accomplished by means of CD3 and CD8 antibodies, which were employed to label cell surface expression of CD3 and CD8. Subsequent to this, fixation and permeabilization were conducted using the Intracellular Fixation & Permeabilization Buffer Set (eBioscience, 88‐8824‐00), followed by using the IFNγ antibody. The stained cells were then analyzed by a FACSCanto II flow cytometer, and the data were further analyzed using Flow Jo 10.0 software.

### 4.17 Immunohistochemistry (IHC)

Tissue samples fixed in formalin and then embedded in paraffin were collected for analysis. The tissue slides were then subjected to an overnight incubation at 65°C, followed by a triple dewaxing process in xylene (15 min each) and a rehydration process in an alcohol gradient. Antigen retrieval was subsequently performed using a citrate buffer solution (from Sangon Biotech), and endogenous peroxidase activity was blocked at 37°C for 30 min. Thereafter, the slides were blocked for 10 min, followed by an overnight 4°C incubation with primary antibodies, and then a 1 h incubation at room temperature with secondary antibodies. Staining was then achieved using the GTVisionTM III Detection System/Mo&Rb (GeneTech, Shanghai, China) following the manufacturer's protocol. The primary antibodies employed in the IHC analysis are listed as follows: UGDH (AG3528, diluted 1:200, Beyotime), p‐UGDH (diluted 1:200), UXS1 (GTX120038, diluted 1:200, Genetex).

To quantify the IHC results, four random fields of view per slide were assessed for staining intensity and coverage. The staining intensity was then assigned a numerical value ranging from 0 (no staining) to 3 (strong staining), while the staining area was scored from 0 (≤5%) to 4 (>75%) of positive cells. The histological score, which is the primary endpoint of this study, was then determined by multiplying these staining intensity and coverage area scores. This score was subsequently categorized into four distinct groups: negative (score 0), weakly positive (+, score 1–4), moderately positive (++, score 5–8), and strongly positive (+++, score 9–12).

### 4.18 Immunofluorescence Assay

The tissue sections were placed in a repair cassette filled with EDTA antigen repair buffer (pH 9.0) for antigen repair under high temperature and pressure. The primary antibodies employed included CD8 (ab209775, dilution 1:4000, abcam), IFNγ (DF6045, dilution 1:800, affinity), CD163 (ab182422, dilution 1:4000, abcam), CD68 (ab201340, dilution 1:100, abcam), F4‐80 (70076, dilution 1:1000, Cell Signaling Technology) and CD206 (24595, dilution 1:200, Cell Signaling Technology). The secondary antibodies employed in this study included donkey anti‐rabbit IgG (H+L) Highly Cross‐Adsorbed Secondary Antibody, Alexa Fluor 594 (A21207, dilution 1:600, ThermoFisher), donkey anti‐mouse IgG (H+L) Highly Cross‐Adsorbed Secondary Antibody, Alexa Fluor 488 (A21202, dilution 1:400, ThermoFisher), and goat anti‐rabbit IgG H&L (HRP) (ab205718, dilution 1:4000, Abcam). Subsequently, the TSA fluorescent dye reaction was performed by washing three times with PBS for 4 min each time. The circles were then incubated with TSA working solution iFluor 488 tyramide (11066, dilution: 1/400, atbio) for 10 min. The slides were then stained with DAPI (Invitrogen; Thermo Fisher Scientific, Inc.) to observe the nuclei. The immunolabeled slides were then viewed using a fluorescence microscope with multiple objective lenses (Leica Microsystem GmbH).

### 4.19 Tumor Organoid Generation

As previously reported [40, 41], immediately following the surgical specimen's delivery, a portion of the tumor tissue and a section of a non‐affected lung tissue (0.5–2 cm^3^) were extracted. A small portion of the surgical specimen was preserved in cold 10% formalin and transferred to the pathology laboratory for confirmation of the presence of non‐small cell lung cancer (NSCLC). The remaining tissue samples were transferred into a liquid medium containing Hank's balanced salt solution (HBSS), which contains an antibiotic and antimycotic agent (15,240,096, ThermoFisher). This step was executed within 12 h post‐surgery. In summary, the tumor samples were minced into small pieces and digested with collagenase type II and DNase I at 37°C. The mixture was stirred continuously at 120 rpm. Subsequently, trypsin was employed to digest the samples for a duration of 10 min. Following this step, the cell mixture was filtered through a 70 µm strainer to remove any large pieces of tissue. The filtered cells were then gently mixed with Matrigel, and 30–50 µL droplets were placed on a 6‐well plate that had been heated to the appropriate temperature. The droplets were then inverted for 1 min and subsequently left for 10 min in an incubator to solidify. Each well was filled with 2.5 mL of warm organoid culture medium, and any empty wells were filled with sterile phosphate‐buffered saline (PBS) to prevent the medium from evaporating. The organoids were cultivated until they reached a diameter of 100–150 µm (which took approximately two weeks). Thereafter, they were analyzed further.

### 4.20 Cell‐Derived Xenograft Models (CDX)

Male BALB/c nude mice, aged 4 weeks, were procured and maintained as previously described [40, 41]. The combination of metformin and plantainoside treatment was then investigated: 5 × 10^6^ A549 cells were mixed, resuspended in 150 µL PBS, and subcutaneously injected into the right flank of each nude mouse. The injection was performed one week after implantation. Mice were divided into four groups, with six mice per group. The groups were treated with 1) 0.9% sodium chloride solution (0.9% NS), 2) metformin (20 mg/kg, dissolved in 0.9% NS), 3) plantainoside (25 mg/kg, dissolved in 100 µL 0.9% NS per mouse), 4) metformin (20 mg/kg) and plantainoside (25 mg/kg, dissolved in 100 µL 0.9% NS per mouse) by intratumoral multiple‐point injection. The mice were treated every three days for a total of nine times. The tumor volume was calculated as (length × width^2^)/2. The xenograft tumors were collected for further analysis 28 days after implantation.

### 4.21 Spontaneous Lung Cancer Model

Six‐week‐old male C57BL/6 mice carrying a Cre‐LoxP‐mediated G12D mutant Kras (Shanghai Model Organisms Center) were utilized. These mice were intranasally administered 5 × 10^10^ copies of the aforementioned AAV. The viral vector utilized was Scgb1a1‐Cre adeno‐associated virus (AAV) (GENECHEM). The system effectively mediated the excision of stop elements, resulting in the activation of oncogenic KRAS expression and subsequent lung tumorigenesis. A two‐month period after AAV administration was followed by CT scanning for the confirmation of lung cancer in these mice. The groups were treated either with 1) 0.9% sodium chloride solution (0.9% NS), 2) metformin (20 mg/kg, dissolved in 0.9% NS), 3) plantainoside (25 mg/kg, dissolved in 100 µL 0.9% NS per mouse), 4) metformin (20 mg/kg) and plantainoside (25 mg/kg, dissolved in 100 µL 0.9% NS per mouse) by intravenous injection only, or corresponding with injection of cisplatin (3 mg/kg) in combination with anti‐PD‐1 pembrolizumab (100 µg per mouse) or the corresponding vehicle every 4 weeks for a total of 16 weeks (4 times if survived at last). To assess the progression of lung cancer, in this study, CT scans were conducted every 4 weeks using a Rigaku Corporation machine (Tokyo, Japan).

### 4.22 Quantification and Statistical Analysis

The results were presented as mean ± standard deviation, with statistical analysis and data visualization executed using R (Version 4.1.1) and GraphPad Prism 9. Either an unpaired Student's *t*‐test or one‐way ANOVA with Bonferroni correction was employed to assess continuous variables depending on the context. The IC50 was calculated using three‐parameter dose‐response curve models. All statistical tests were two‐tailed, and a *p*‐value of less than 0.05 was considered to be significant. All analyses were performed blindly, such that experimenters performing IHC, anatomical measures, and data analysis were unaware of the groups and treatments.

### 4.23 Bioinformatics Analysis Methods and Parameters

Metabolomic analyses were conducted across three biological contexts: mouse tumor tissues (n = 6/group), patient‐derived tumors (n = 6/group), and LUAD cell lines (n = 6/group). Differential metabolite screening employed stringent thresholds tailored to each system: |log2 fold change (FC)| >1 with *p*< 0.05 for mouse tissues and cell lines, while patient tumors required |log2FC| >2 with *p*< 0.01 to prioritize clinically relevant alterations.

Single‐cell RNA sequencing (scRNA‐seq) data from patient tumors underwent rigorous preprocessing via Seurat (v5.0), including normalization, log‐transformation, and selection of the top variable genes. Cell clusters were annotated using CellMarker 2.0, revealing canonical lineages.

Survival associations were evaluated using the Kaplan‐Meier (KM) plotter (http://kmplot.com), correlating metabolite/gene expression levels with overall survival in TCGA‐LUAD cohorts. Immune infiltration patterns were quantified with CIBERSORTx, leveraging the LM22 signature matrix to estimate tumor microenvironment composition.

## Funding Statement

This work was supported by grants from the China Postdoctoral Innovative Talent Support Program (No. BX20250246), the China Postdoctoral General Funding Program (No. 2025M7722227), the Shanghai Sailing Program (No. 24YF2731100), the National Natural Science Foundation of China (Nos. 82473184 and 82303442), and the Youth Foundation of the Zhongshan Hospital, Fudan University (No. ZSZP202414).

## Ethics Statement

All methods were carried out in accordance with relevant guidelines and regulations. All participants of the clinical study signed informed consent according to the ethical requirements in the Declaration of Helsinki, which was approved by the Ethics Committee of Zhongshan Hospital, Fudan University (B2021–267). All animal experiments complied with the WMA Statement on animal use in biomedical research and EU recommendations (Directive 2010/63/EU) for experimental design and analysis in pharmacology care, and animal experiments were approved by the Ethics Committee of Zhongshan Hospital, Fudan University (2022–203).

## Consent

Written informed consent to participate and to publish was obtained from participants before inclusion in the study.

## Conflicts of Interest

The authors declare no conflicts of interest.

## Supporting information




**Supporting File 1**: advs75653‐sup‐0001‐TableS1.docx.

**Supporting File 2**: advs75653‐sup‐0002‐TableS2.docx.

**Supporting File 3**: advs75653‐sup‐0003‐TableS3.docx.

**Supporting File 4**: advs75653‐sup‐0004‐TableS4.xlsx.

**Supporting File 5**: advs75653‐sup‐0005‐SuppMat.docx.

**Supporting File 6**: advs75653‐sup‐0006‐Data.docx.

## Data Availability

The data that support the findings of this study are available from the corresponding author upon reasonable request.
