# Supplementary material for: Targeting UXS1‐Dependent Glucuronate Detoxification Potentiates Metformin's Anti‐Tumor Efficacy in Lung Adenocarcinoma
Source: Adv Sci (Weinh). 2026 May 10;13(44):e10542. doi: 10.1002/advs.202510542 (PMC13336104; doi:10.1002/advs.202510542)
Supplement: Supplementary file 4 [file ADVS-13-e10542-s005.docx]

**Supplementary Table 2 Details of LUAD patients’ samples used for the organoid models**

|  | Sex* | Age | Smoking History | | Tumor Site* | p-T | p-N | M | Tumor Size (mm) | p-stage | Gene Mutation | Status |
| --- | --- | --- | --- | --- | --- | --- | --- | --- | --- | --- | --- | --- |
| NC-1 | F | 55 | No | RUL | | 1c | 1 | 0 | 21*11*3.5 | IIB | EGFR 19del | survival |
| NC-2 | M | 66 | No | RUL | | 2a | 0 | 0 | 38*18*15 | IB | TP53 | survival |
| NC-3 | F | 60 | No | LLL | | 1c | 0 | 0 | 29*15*11 | IA3 | ALK | survival |
| NC-4 | F | 56 | No | LUL | | 2a | 1 | 0 | 20*30*25, PL1 | IIB | EGFR 19del, TP53 | survival |
| NC-5 | M | 71 | Yes | RML | | 2a | 0 | 0 | 34*25*20 | IB | None | survival |
| Met-1 | M | 74 | Yes | LUL | | 2a | 0 | 0 | 35*25*30 | IB | Met | survival |
| Met-2 | F | 56 | No | LLL | | 1b | 0 | 0 | 20*20*10 | IA2 | EGFR 19del | survival |
| Met-3 | M | 82 | No | RUL | | 2a | 0 | 0 | 35*20*20 | IB | KRAS G12C | survival |
| Met-4 | F | 43 | No | LUL | | 1b | 0 | 0 | 12*8*6 | IA2 | EFGR L858R | survival |
| Met-5 | M | 68 | No | RLL | | 1c | 0 | 0 | 30*11*10 | IA3 | None | survival |

* F: Female; M: Male

** RUL: Right Upper Lung; RLL: Right Lower Lung; LUL: Left Upper Lung; LLL: Left Lower Lung
