# Supplementary material for: Targeting UXS1‐Dependent Glucuronate Detoxification Potentiates Metformin's Anti‐Tumor Efficacy in Lung Adenocarcinoma
Source: Adv Sci (Weinh). 2026 May 10;13(44):e10542. doi: 10.1002/advs.202510542 (PMC13336104; doi:10.1002/advs.202510542)
Supplement: Supplementary file 5 [file ADVS-13-e10542-s006.docx]

**Supplementary Table 1 Details of the three common differential metabolites upon metformin treatment across the three models**

|  | **Metabolites** | **log2 Fold Change** | **Regulation** | **p-value** |
| --- | --- | --- | --- | --- |
| Animal model | Serylhistidine | 5.91359 | Up | 2E-05 |
|  | Octadecyl Fumarate | 4.14579 | Up | 0.04955 |
|  | Uridine Diphosphate Glucose | -1.1475 | Down | 0.01616 |
| Tissue collection | Serylhistidine | ‌2.02208 | Down | 0.00029 |
|  | Octadecyl Fumarate | 2.55181 | Up | 0.00032 |
|  | Uridine Diphosphate Glucose | -2.37987 | Down | 0.00034 |
| Cell Culture | Octadecyl Fumarate | 1.79195 | Up | 0.03015 |
|  | Serylhistidine | 1.09246 | Up | 0.02844 |
|  | Uridine Diphosphate Glucose | -1.46638 | Down | 0.03258 |
