# Supplementary material for: Targeting UXS1‐Dependent Glucuronate Detoxification Potentiates Metformin's Anti‐Tumor Efficacy in Lung Adenocarcinoma
Source: Adv Sci (Weinh). 2026 May 10:e10542. Online ahead of print. doi: 10.1002/advs.202510542 (PMC13336104; doi:10.1002/advs.202510542)
Supplement: Supplementary file 3 — Supporting File 3: advs75653‐sup‐0003‐TableS3.docx. [file ADVS-9999-e10542-s006.docx]

**Supplementary Table 3 The sequences and melting temperature (Tm) of the primers used in our research, whether they span exon junctions**

| Gene | Primers Sequence 5’-3’ | Tm (℃) | Exon junction span |
| --- | --- | --- | --- |
|  | (forward, reverse) |  |  |
| UGDH | GCTCGTTATTGGCAGCAGGTCA | 59.6 | Yes |
|  | CGGGACACTTGGTCATCCTCTG | 58.7 |  |
| UGP2 | TCTGGATCTGACTGTTCAGCA | 60.5 | Yes |
|  | TGAGTAAGACACGTCCTTTGC | 60 |  |
| UXS1 | GTTGCAGAGACCATGTGCTAT | 60.1 | Yes |
|  | CTACTCGCCCATCGTTCATGT | 61.9 |  |
| CD206 | CTGCCAACAACAGAACGCTGAG | 58.7 | Yes |
|  | TCCAATCCAGAGTCCTGAGGTCAA | 59.2 |  |
| CD163 | TTTGTCAACTTGAGTCCCTTCAC | 60.6 | Yes |
|  | TCCCGCTACACTTGTTTTCAC | 60.5 |  |
| CD86 | ACGACGTTTCCATCAGCTTGTCT | 59 | Yes |
|  | TGGTCTGTTCACTCTCTTCCCTCT | 58.9 |  |
| CD80 | CCAACCACAGCTTCATGTGTCTCA | 59.8 | Yes |
|  | AGCAGTAGGTCAGGCAGCATATCA | 60 |  |
| IFNγ | TCGGTAACTGACTTGAATGTCCA | 61.2 | Yes |
|  | TCGCTTCCCTGTTTTAGCTGC | 62.9 |  |
| ACTB | CATGTACGTTGCTATCCAGGC | 60.8 | Yes |
|  | CTCCTTAATGTCACGCACGAT | 60.2 |  |
