# Supplementary material for: Targeting UXS1‐Dependent Glucuronate Detoxification Potentiates Metformin's Anti‐Tumor Efficacy in Lung Adenocarcinoma
Source: Adv Sci (Weinh). 2026 May 10:e10542. Online ahead of print. doi: 10.1002/advs.202510542 (PMC13336104; doi:10.1002/advs.202510542)
Supplement: Supplementary file 5 — Supporting File 5: advs75653‐sup‐0005‐SuppMat.docx. [file ADVS-9999-e10542-s003.docx]

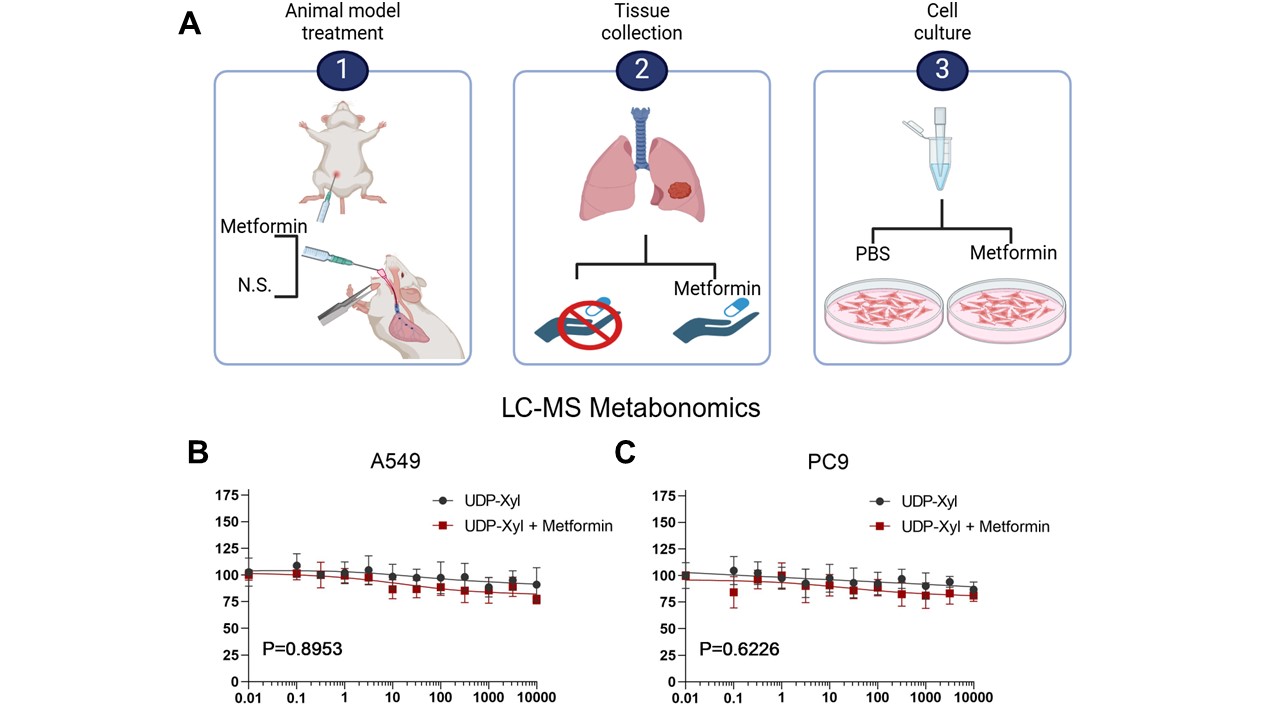


**Supplementary Figure 1.** (A) Flowchart illustrating the experimental design and workflow for metabolomic sequencing. The analysis includes subcutaneous tumors from nude mice treated with or without metformin (n = 6 mice per group), tumor specimens from LUAD patients with or without metformin intake (n = 6 patients per group), and the LUAD cell line A549 cultured with or without metformin (n = 6 biologically independent samples per group). (B, C) CCK-8 cell viability assays evaluating the effects of UDP-Xyl treatment, in the presence or absence of metformin, on A549 (B) and PC9 (C) cells (n = 3 independent experiments). Statistical information: Statistical significance was determined using one-way or two-way ANOVA followed by Bonferroni's post hoc test for multiple comparisons in panels B and C.


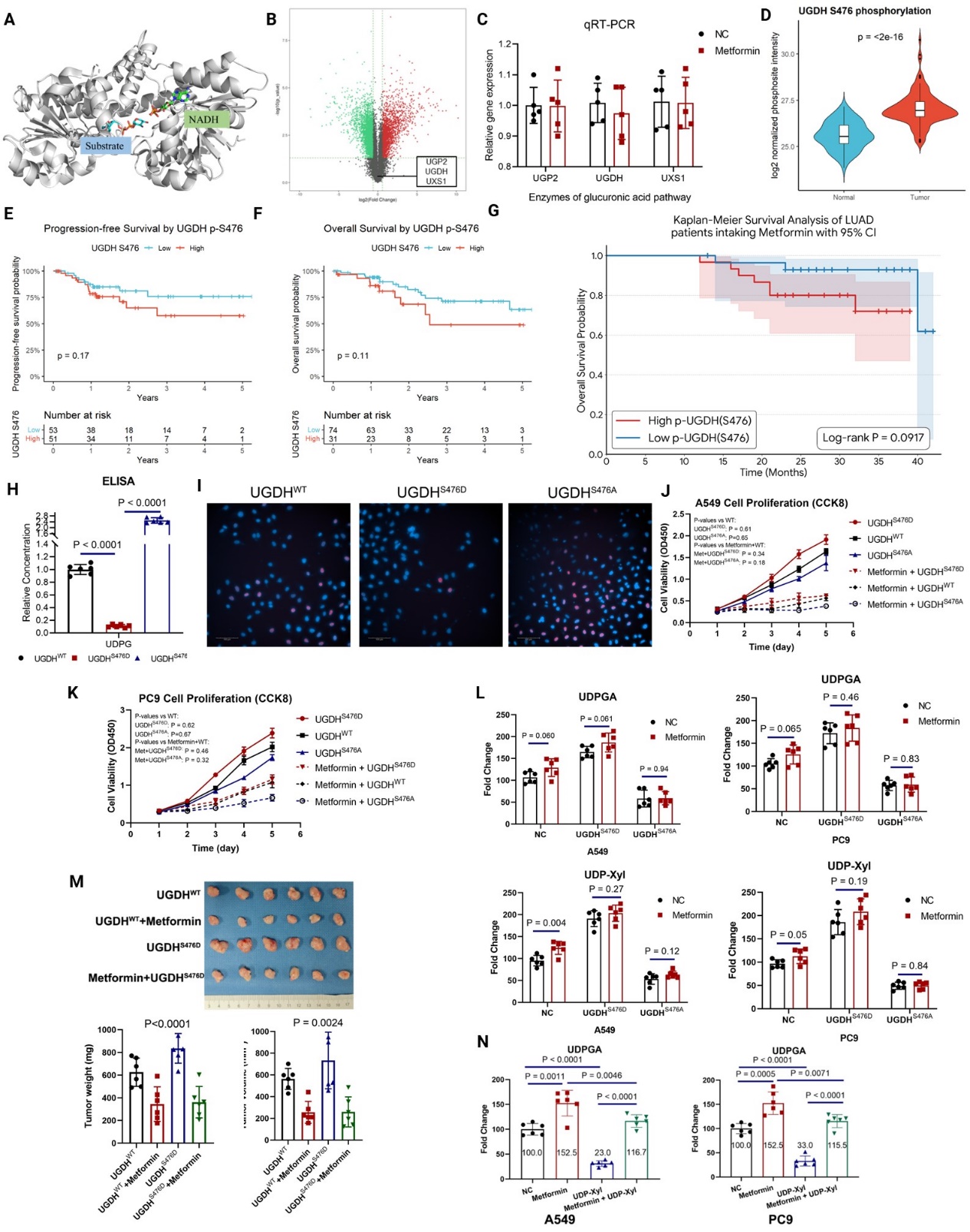


**Supplementary Figure 2.** (A) 3D structural model of the UGDH protein. (B) Volcano plot displaying the transcriptome sequencing results comparing LUAD cells cultured with or without metformin (n = 3 biologically independent samples per group). (C) qRT-PCR analysis verifying the lack of significant differences in the mRNA expression levels of UGP2, UGDP, and UXS1 in LUAD cells cultured with or without metformin (n = 3 independent experiments). (D) Analysis of LUAD phosphoproteomics data from the CPTAC database, confirming that the phosphorylation level of UGDH S476 is significantly upregulated in LUAD tumor tissues (n = 110) compared to normal tissues (n = 104) (*P* < 2e-16). (E, F) Kaplan-Meier survival analysis illustrating the relationship between p-UGDH(S476) levels and patient prognosis in the CPTAC cohort, showing Overall Survival (OS) (E) and Progression-Free Survival (PFS) (F). Patients were stratified into high and low phosphorylation groups using the optimal cut-off approach (*P* = 0.11 for OS; *P* = 0.17 for PFS). (G) Kaplan-Meier overall survival (OS) analysis of a strictly controlled retrospective cohort of 60 LUAD patients (using surgical specimens collected in 2022), stratified by high versus low UGDH S476 phosphorylation levels. The Log-rank test revealed a trend of better survival in the low phosphorylation group (*P* = 0.0917). (H) ELISA quantification verifying the changes in UDPG levels among UGDH-KO LUAD cells re-expressing UGDH^WT^, UGDH^S476A^ and UGDH^S476D^ (n = 3 independent experiments). (I) EdU cell proliferation assays evaluating UGDH-KO LUAD cells re-expressing UGDH^WT^, UGDH^S476A^ and UGDH^S476D^ (n = 3 independent experiments). (J, K) CCK-8 assays evaluating the cell proliferation capacity of A549 (J) and PC9 (K) cells. Cells stably expressing UGDH^WT^, the phospho-dead mutant UGDH^S476A^, or the phospho-mimetic mutant UGDH^S476D^ were cultured in the presence or absence of Metformin and monitored continuously over a 5-day period (n = 3 independent samples per group). (L) Targeted metabolomics analyses demonstrating the intracellular levels of UDPGA and UDP-Xyl in cells expressing the S476A and S476D mutants, treated with or without metformin (n = 3 biologically independent samples per group). (M) Representative images and/or tumor weight/volume analysis of subcutaneous xenograft tumors derived from cells harboring the S476D mutation, treated with or without metformin (n = 6 mice per group). (N) Targeted metabolic mass spectrometry detecting the effects of combined metformin and UDP-Xyl treatment on the intracellular content of UDPGA in A549 and PC9 cells (n = 3 biologically independent samples per group).


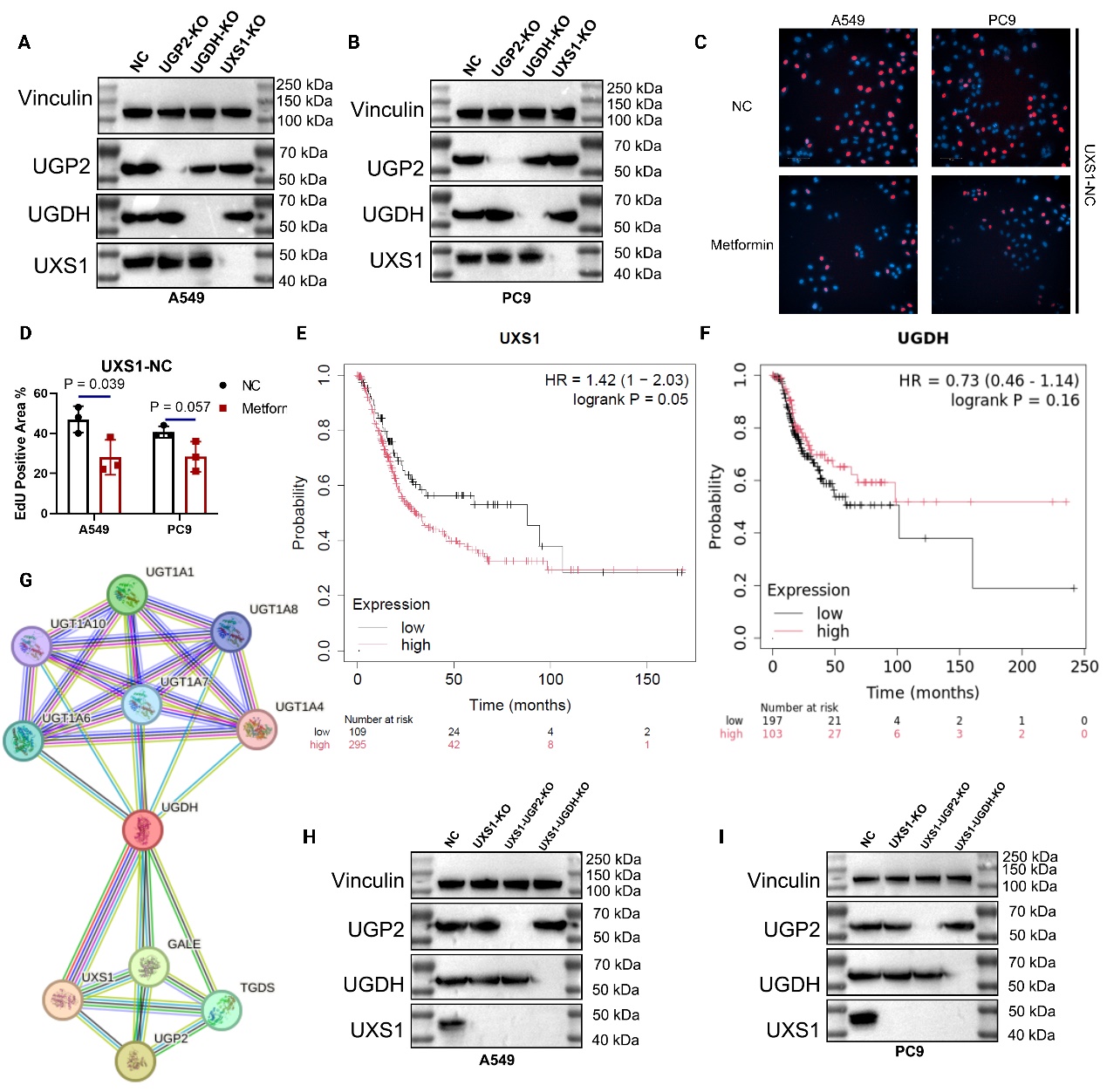


**Supplementary Figure 3**. (A, B) Representative Western blot images verifying the successful knockout of UGP2, UGDH, and UXS1 (UGP2-KO, UGDH-KO, and UXS1-KO) in A549 (A) and PC9 (B) cells compared to control cells (n = 3 independent experiments). (C, D) EdU cell proliferation assays evaluating the effect of metformin treatment on the proliferation capacity of negative control (UXS1-NC) A549 and PC9 cells (n = 3 independent experiments). (E) Kaplan-Meier survival analysis demonstrating that LUAD patients with high UGDH expression exhibited a better prognosis (n = 404 patients). (F) Kaplan-Meier survival analysis revealing that LUAD patients with high UXS1 expression had a poorer prognosis (n = 300 patients). (G) Protein-protein interaction (PPI) network analysis illustrating that UGDH is closely associated with UXS1 and UGP2. (H, I) Representative Western blot images confirming the successful secondary knockout of UGP2 or UGDH in the UXS1-KO background (double knockout) in A549 (H) and PC9 (I) cells (n = 3 independent experiments). Statistical information: Statistical significance was determined using the two-tailed unpaired Student's t-test for comparisons between two groups in the EdU assays (panels C, D). The survival differences in panels E and F were assessed using the Log-rank test.


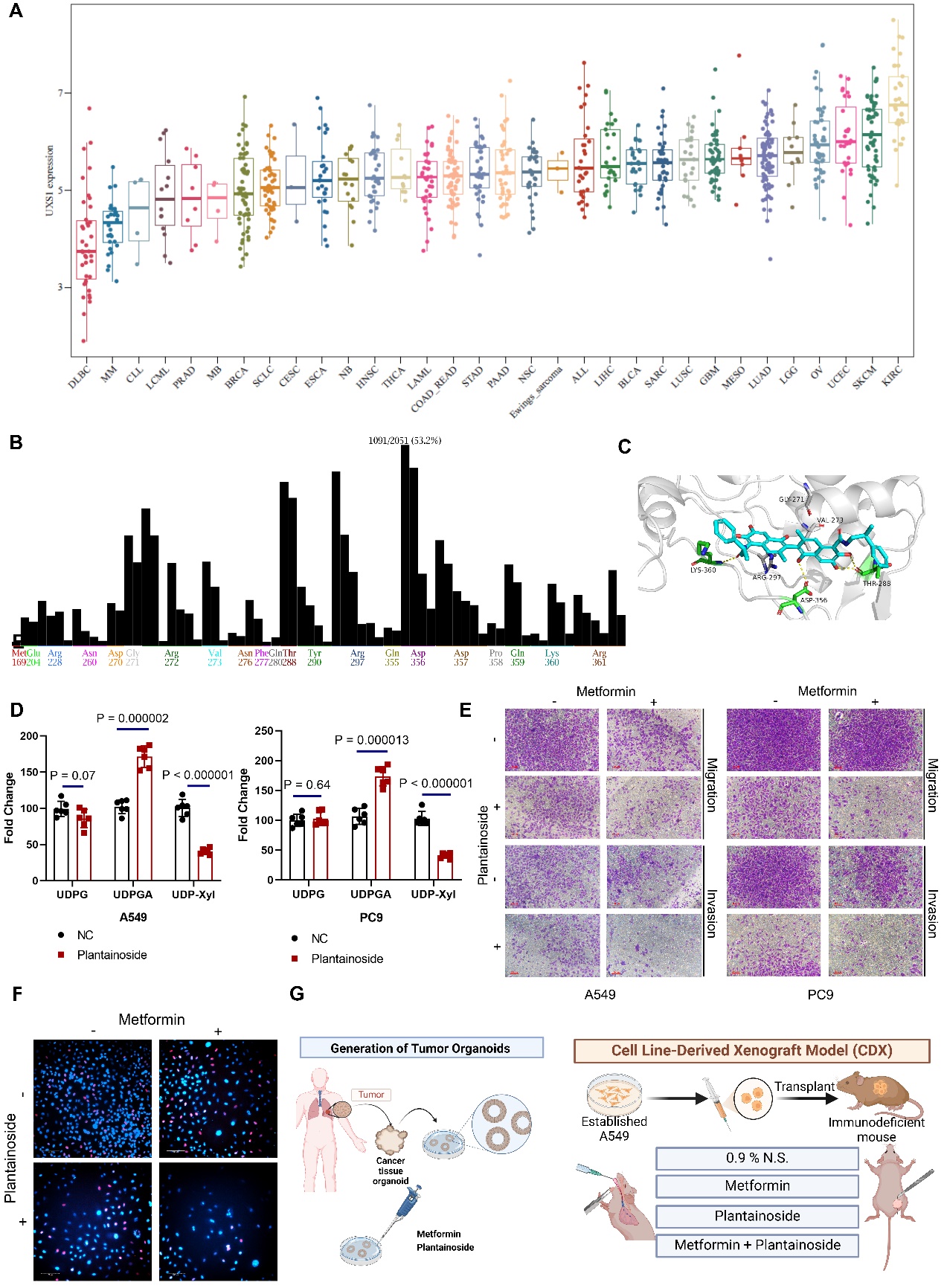


**Supplementary Figure 4.** (A) Analysis of UXS1 expression levels across various tumor cell lines using the CCLE (Cancer Cell Line Encyclopedia) database. (B) Protein-Ligand Interaction Fingerprint (PLIF) analysis of the UXS1 protein with virtual screening hit molecules. Identical amino acid residues are indicated by the same color, and a higher vertical density represents a higher frequency of interaction between the residues and the hit compounds. (C) 3D structural model illustrating the molecular docking and binding interactions between plantainoside and UXS1. (D) metabolomics analysis evaluating the effects of plantainoside treatment in wild-type (WT) versus UGDH-knockout (UGDH-KO) A549 and PC9 cells (n = 6 independent experiments). The data demonstrate a genetic rescue effect confirming that the drug's toxicity is dependent on upstream UDPGA metabolic flux. (E) Transwell assays evaluating the individual and combined effects of plantainoside and metformin (1 mM) on the migration and invasion capacities of LUAD cells (n = 3 independent experiments). (F) EdU cell proliferation assays comparing the effects of plantainoside alone, metformin (1 mM) alone, and their combination on the proliferation of LUAD cells (n = 3 independent experiments). (G) Schematic flow chart illustrating the experimental protocol for the organoid culture model. (H) Schematic flow chart depicting the experimental design and treatment timeline for the Cell Line-Derived Xenograft (CDX) tumor model in nude mice. Statistical information: Statistical significance was determined using one-way or two-way ANOVA followed by Bonferroni's post hoc test for multiple group comparisons (panels D, E, F).


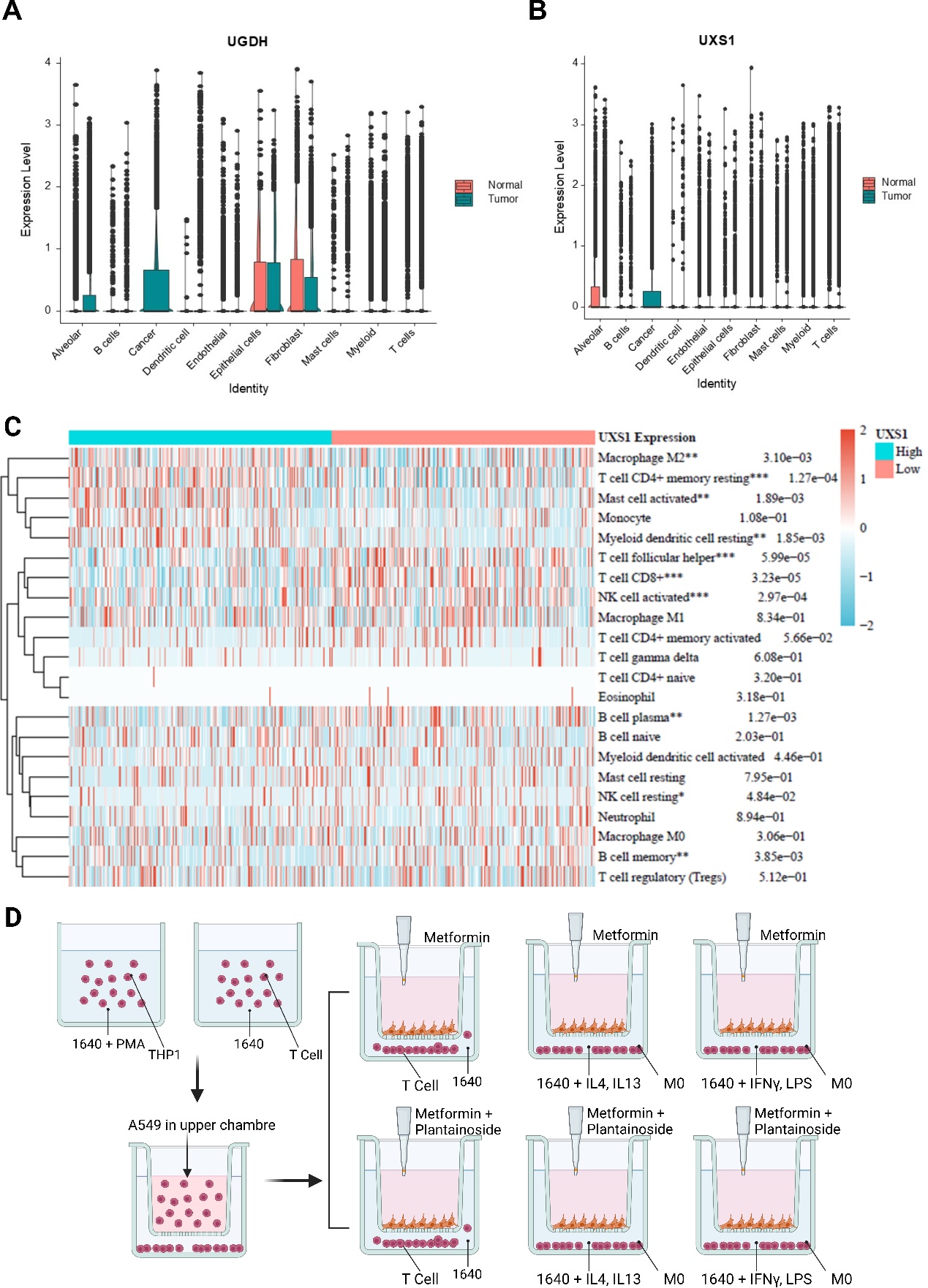


**Supplementary Figure 5.** (A, B) Single-cell RNA sequencing (scRNA-seq) analysis demonstrating the predominant expression of UGDH (A) and UXS1 (B) within the tumor cell populations of LUAD patients receiving metformin treatment. Violin plots were used to visualize the cell clustering and gene expression distribution. (C) Heatmap illustrating the differential immune cell infiltration landscape between LUAD patients with high versus low UXS1 expression in the TCGA database. The relative abundance of tumor-infiltrating immune cells was estimated using the CIBERSORT algorithm (n = 516 patients per group). (D) Schematic diagram illustrating the experimental workflow for the *in vitro* co-culture system, detailing the interaction between LUAD cells and immune cells (T cells and macrophages) following treatment with metformin alone or in combination with plantainoside.
