# Supplementary figures and images for: Targeting UXS1‐Dependent Glucuronate Detoxification Potentiates Metformin's Anti‐Tumor Efficacy in Lung Adenocarcinoma
Source: Adv Sci (Weinh). 2026 May 10:e10542. Online ahead of print. doi: 10.1002/advs.202510542 (PMC13336104; doi:10.1002/advs.202510542)

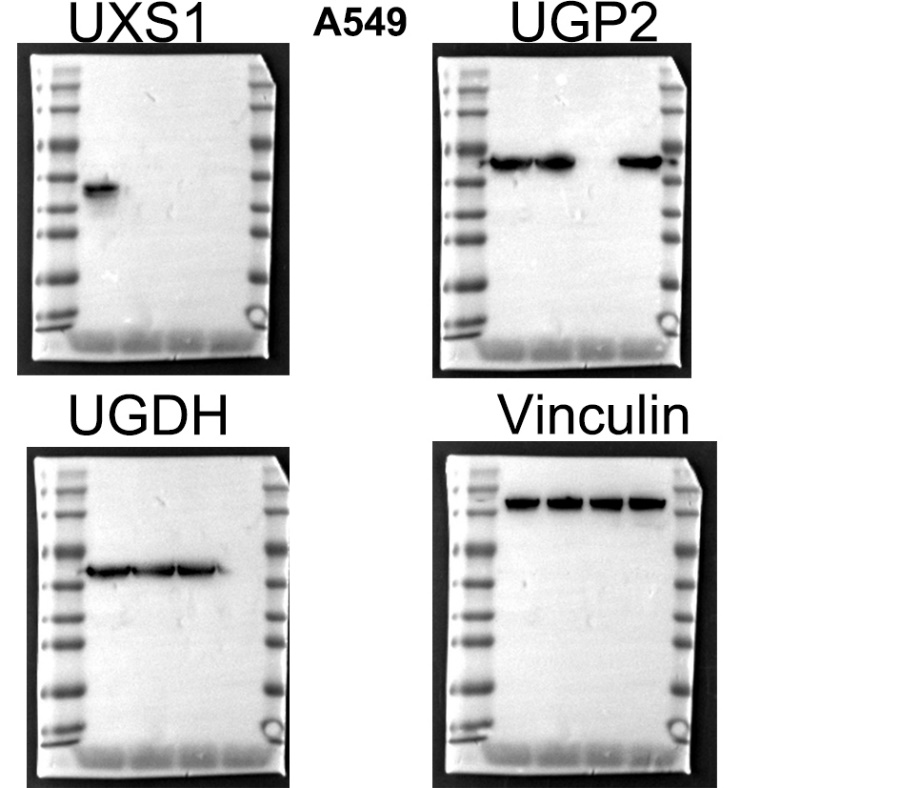

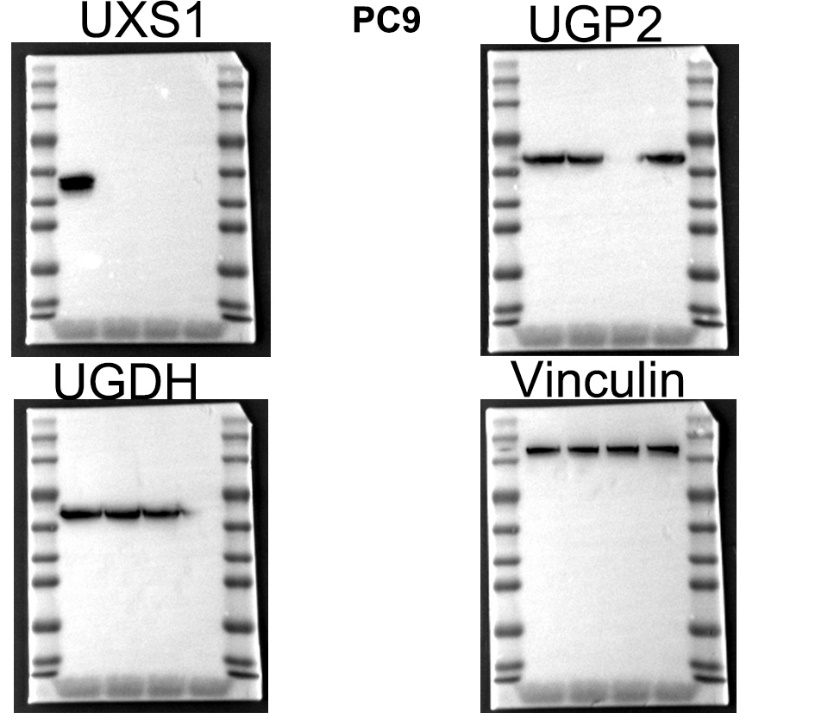

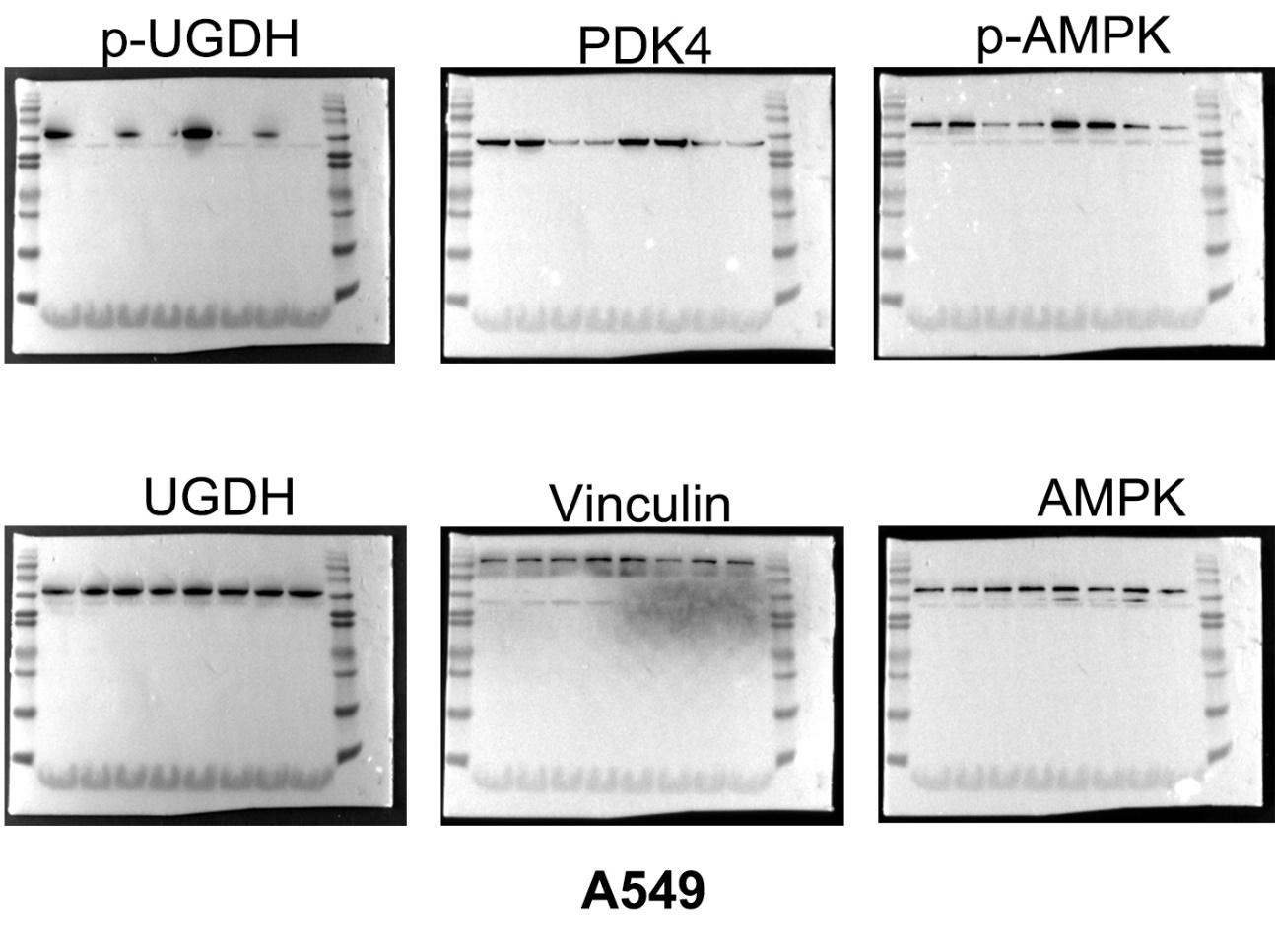

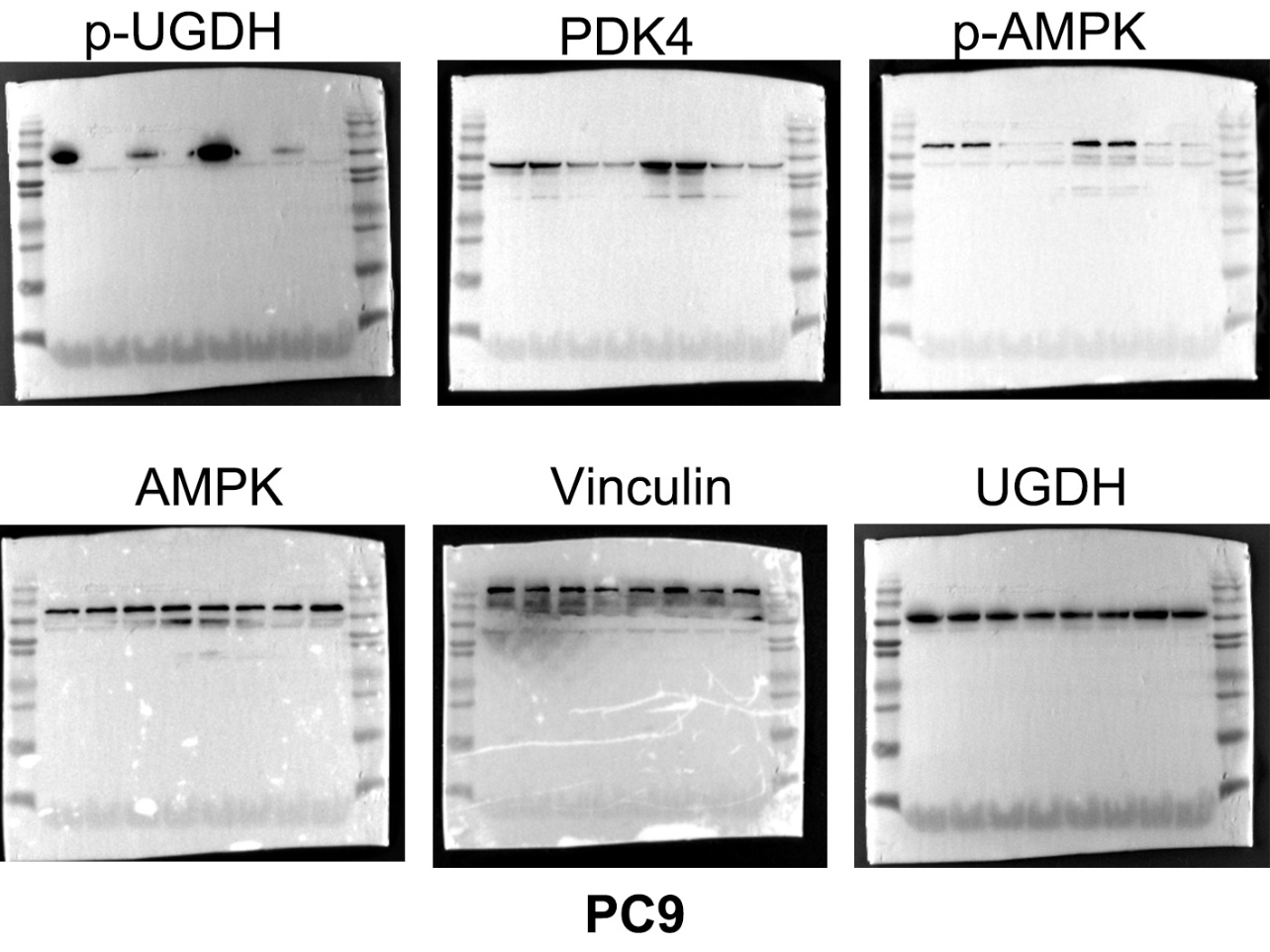


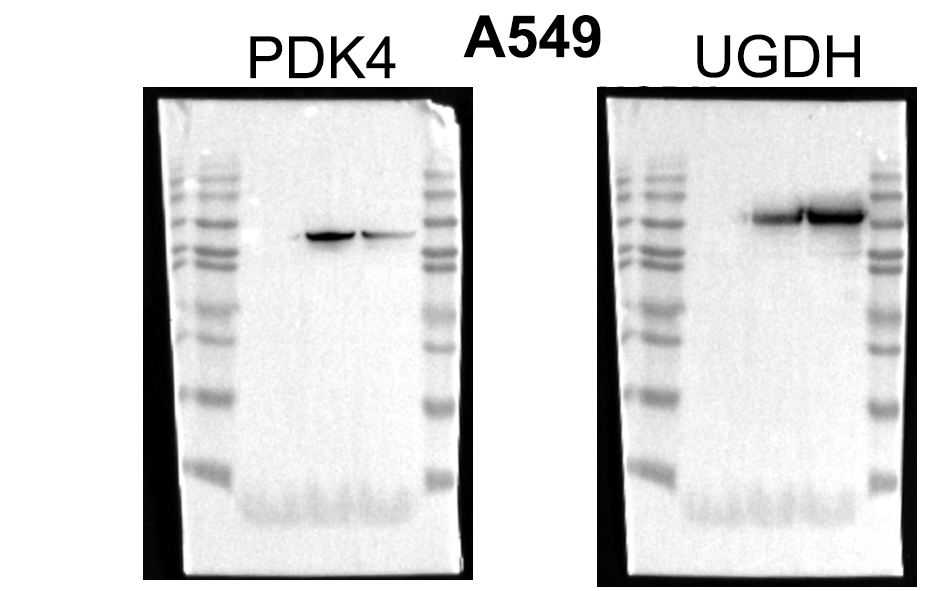


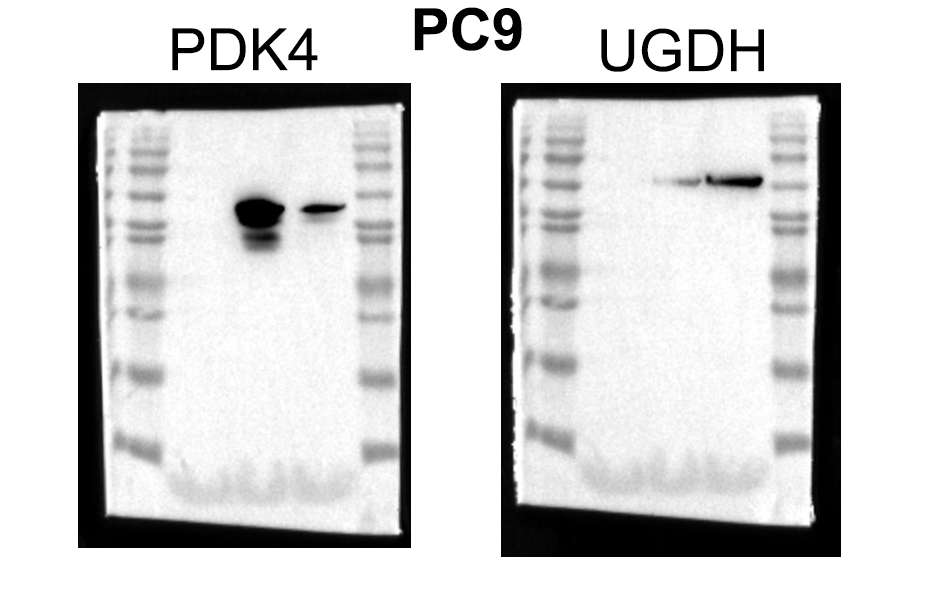


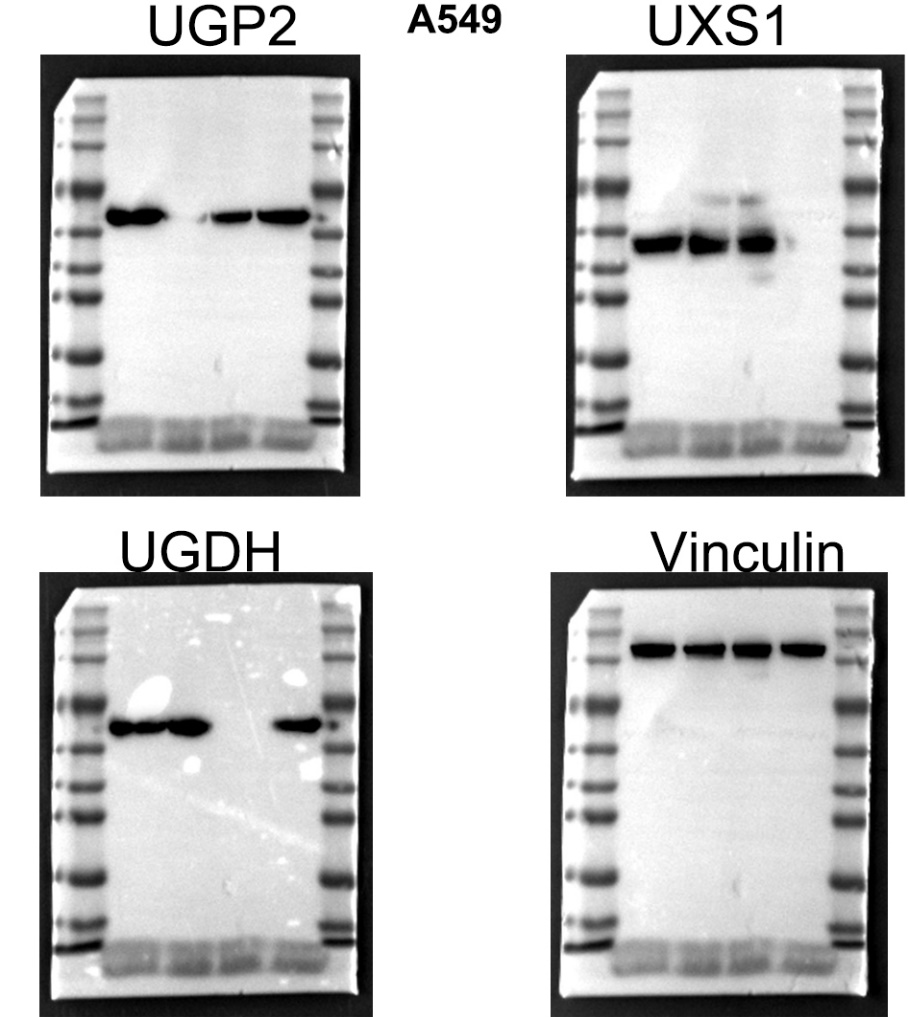


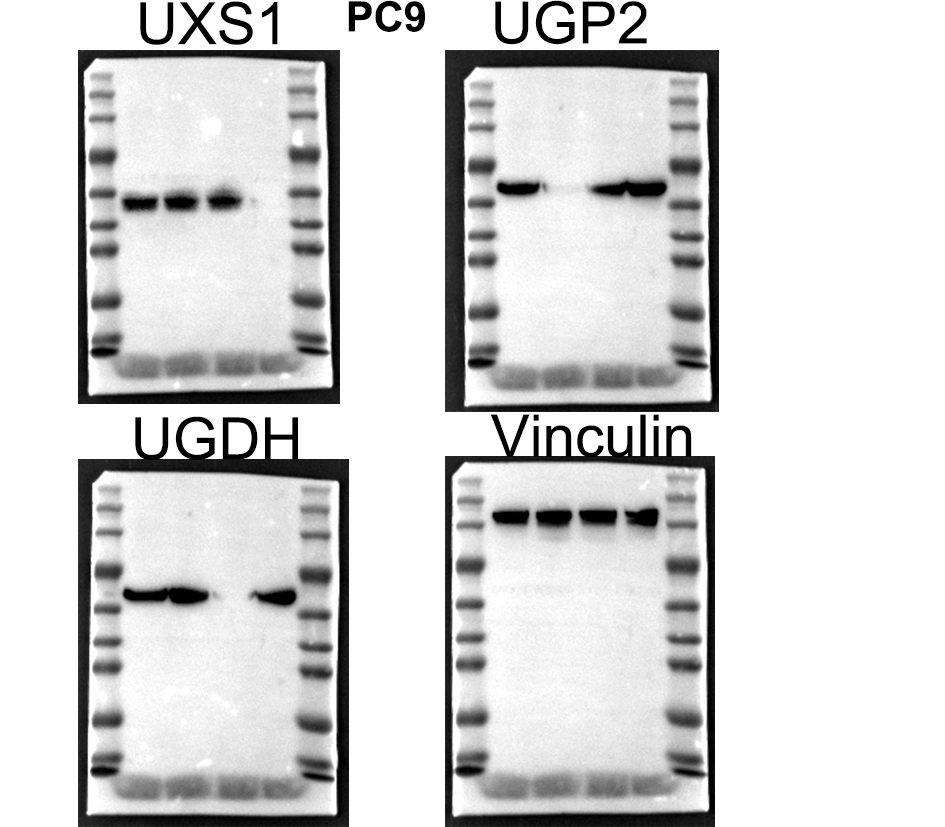

Supplement: Supplementary file 6 — Supporting File 6: advs75653‐sup‐0006‐Data.docx. [file ADVS-9999-e10542-s004.docx]
